# The Long-Term Safety, Public Health Impact, and Cost-Effectiveness of Routine Vaccination with a Recombinant, Live-Attenuated Dengue Vaccine (Dengvaxia): A Model Comparison Study

**DOI:** 10.1371/journal.pmed.1002181

**Published:** 2016-11-29

**Authors:** Stefan Flasche, Mark Jit, Isabel Rodríguez-Barraquer, Laurent Coudeville, Mario Recker, Katia Koelle, George Milne, Thomas J. Hladish, T. Alex Perkins, Derek A. T. Cummings, Ilaria Dorigatti, Daniel J. Laydon, Guido España, Joel Kelso, Ira Longini, Jose Lourenco, Carl A. B. Pearson, Robert C. Reiner, Luis Mier-y-Terán-Romero, Kirsten Vannice, Neil Ferguson

**Affiliations:** 1 London School of Hygiene and Tropical Medicine, London, United Kingdom; 2 Johns Hopkins Bloomberg School of Public Health, Baltimore, Maryland, United States of America; 3 Sanofi Pasteur, Lyon, France; 4 University of Exeter, Exeter, United Kingdom; 5 Duke University, Durham, North Carolina, United States of America; 6 University of Western Australia, Crawley, Australia; 7 University of Florida, Gainesville, Gainesville, Florida, United States of America; 8 University of Notre Dame, Notre Dame, Indiana, United States; 9 Imperial College London, London, United Kingdom; 10 University of Oxford, Oxford, United Kingdom; 11 Indiana University, Bloomington, Indiana, United States of America; 12 World Health Organization, Geneva, Switzerland; Mahidol-Oxford Tropical Medicine Research Unit, THAILAND

## Abstract

**Background:**

Large Phase III trials across Asia and Latin America have recently demonstrated the efficacy of a recombinant, live-attenuated dengue vaccine (Dengvaxia) over the first 25 mo following vaccination. Subsequent data collected in the longer-term follow-up phase, however, have raised concerns about a potential increase in hospitalization risk of subsequent dengue infections, in particular among young, dengue-naïve vaccinees. We here report predictions from eight independent modelling groups on the long-term safety, public health impact, and cost-effectiveness of routine vaccination with Dengvaxia in a range of transmission settings, as characterised by seroprevalence levels among 9-y-olds (SP9). These predictions were conducted for the World Health Organization to inform their recommendations on optimal use of this vaccine.

**Methods and Findings:**

The models adopted, with small variations, a parsimonious vaccine mode of action that was able to reproduce quantitative features of the observed trial data. The adopted mode of action assumed that vaccination, similarly to natural infection, induces transient, heterologous protection and, further, establishes a long-lasting immunogenic memory, which determines disease severity of subsequent infections. The default vaccination policy considered was routine vaccination of 9-y-old children in a three-dose schedule at 80% coverage. The outcomes examined were the impact of vaccination on infections, symptomatic dengue, hospitalised dengue, deaths, and cost-effectiveness over a 30-y postvaccination period. Case definitions were chosen in accordance with the Phase III trials.

All models predicted that in settings with moderate to high dengue endemicity (SP9 ≥ 50%), the default vaccination policy would reduce the burden of dengue disease for the population by 6%–25% (all simulations: –3%–34%) and in high-transmission settings (SP9 ≥ 70%) by 13%–25% (all simulations: 10%– 34%). These endemicity levels are representative of the participating sites in both Phase III trials. In contrast, in settings with low transmission intensity (SP9 ≤ 30%), the models predicted that vaccination could lead to a substantial increase in hospitalisation because of dengue. Modelling reduced vaccine coverage or the addition of catch-up campaigns showed that the impact of vaccination scaled approximately linearly with the number of people vaccinated. In assessing the optimal age of vaccination, we found that targeting older children could increase the net benefit of vaccination in settings with moderate transmission intensity (SP9 = 50%). Overall, vaccination was predicted to be potentially cost-effective in most endemic settings if priced competitively.

The results are based on the assumption that the vaccine acts similarly to natural infection. This assumption is consistent with the available trial results but cannot be directly validated in the absence of additional data. Furthermore, uncertainties remain regarding the level of protection provided against disease versus infection and the rate at which vaccine-induced protection declines.

**Conclusions:**

Dengvaxia has the potential to reduce the burden of dengue disease in areas of moderate to high dengue endemicity. However, the potential risks of vaccination in areas with limited exposure to dengue as well as the local costs and benefits of routine vaccination are important considerations for the inclusion of Dengvaxia into existing immunisation programmes. These results were important inputs into WHO global policy for use of this licensed dengue vaccine.

## Abstract 2

Mark Jit and colleagues report findings from eight independent modelling groups on the long-term safety, public health impact, and cost-effectiveness of routine vaccination with a recombinant, live-attenuated dengue vaccine (Dengvaxia).

## Author Summary

**Why Was This Study Done?:**

- Dengvaxia, the first vaccine against all dengue serotypes, has recently been licensed in several countries.
- The World Health Organization initiated this study to inform its official position on the use of Dengvaxia.

**What Did the Researchers Do and Find?:**

- We used dynamical models with differences in how dengue epidemiology is reflected but common assumptions on vaccine effects, informed by trial observations, to predict the impact and cost-effectiveness of Dengvaxia in a variety of situations.
- The results showed that Dengvaxia has the potential to reduce the number of hospitalisations because of dengue by 13%–25% and be cost effective in settings where dengue is common.
- However, in settings with low dengue prevalence, vaccination may increase the incidence of severe illness.

**What Do These Findings Mean?:**

- WHO has now recommended countries consider the use of Dengvaxia in settings with high dengue endemicity.
- Our results can guide countries on the general suitability of Dengvaxia introduction; however, local demographics, heterogeneities in endemicity, and health system costs will need to be taken into account.

## Introduction

Recent estimates indicate that dengue virus (DENV) causes at least 50 million cases of symptomatic disease per year [1,2]. DENV is prevalent across the tropics and subtropics, but transmission intensity is highly spatiotemporally variable. Infection with one of the four serotypes (DENV1–4) appears to provide long-lived immunity to that serotype (homologous protection) and induces temporary cross-reactive immunity to other serotypes (heterologous protection) [3,4]. Because of the short-lived nature of heterologous protection, individuals can experience multiple DENV infections over their lifetimes. The first (primary) DENV infection typically causes no or mild disease, but secondary DENV infection carries a substantially increased risk of severe disease [5], thought to be a result of antibody-dependent enhancement (ADE). Postsecondary infections that result in severe disease are rarely observed and hence believed to be generally mild [4,6,7].

Sanofi Pasteur recently completed Phase III trials of a recombinant, live-attenuated dengue vaccine (CYD-TDV; Dengvaxia) in Latin America and Southeast Asia [8,9] which by late 2016 has been approved in several countries. Over the 25-mo active surveillance phase of the trials, average efficacy against virologically confirmed clinical dengue of a three-dose regimen administered at 6 mo intervals was 60.3% (95% CI: 55.7% to 64.5%) in children between 2 and 16 y. Efficacy varied by age, serotype, and country and was about twice as high in children who were dengue seropositive (i.e., who have had at least one prior infection with any dengue serotype) at the time of vaccination compared to those who were seronegative.

Hospital-based detection of cases has continued and will provide long-term follow-up information during the 4 y following the active phase. The first year of long-term follow-up showed that the vaccine remained protective in all age-groups except among 2- to 5-y-old children. In this age group, vaccinees had a higher incidence of hospitalisation with virologically confirmed DENV than unvaccinated controls [10]. In addition, vaccine efficacy among all age groups was found to be lower during the hospital-based phase than during the active phase, suggesting waning of vaccine-induced protection.

In April 2015, WHO initiated an open call for mathematical modellers to participate in a consortium called “Comparative modelling of dengue vaccine public health impact” (CMDVI) [11,12]. The purpose of the consortium was to generate model-based predictions of the long-term public health impact of Dengvaxia, reflecting the balance of safety, population-level effectiveness, and economic considerations. The participation of multiple modelling groups with transparent assumptions enhances the value of the outputs for policy-making. WHO’s independent expert advisory committee, the Strategic Advisory Group of Experts (SAGE) on Immunization, incorporated the results of this model comparison into their recommendations for appropriate use of the vaccine [13–17]; here, we report our detailed methods, results, and their wider implications.

## Methods

### Consortium Formation

Ten groups responded to the open call by WHO for consortium participation [11,12]. Of these, two groups withdrew subsequently because of time constraints, leaving the following eight groups as participants: Duke University (Duke), University of Exeter/University of Oxford (Exeter/Oxford), Johns Hopkins University/University of Florida (Hopkins/UF), Imperial College London (Imperial), University of Notre Dame (Notre Dame), Sanofi Pasteur, University of Florida (UF), and University of Western Australia (UWA). All groups agreed to run epidemiological scenarios using a common set of assumptions about vaccine action (with varying parameterisation) that were mutually agreed on in discussions with the SAGE Working Group on Dengue Vaccines and Vaccination [18].

### Transmission Models

Details of the transmission models used have been published elsewhere [19–28], but concise descriptions are provided in S1 Appendix. All models simulated infections by all four serotypes and most included cross-protection between serotypes (all except Exeter/Oxford) and explicitly represented vector population dynamics (all except Duke). All models used demographic parameters typical of dengue-endemic middle-income countries (Table 1 and S1 Appendix). Half of the models were deterministic compartmental models (Sanofi Pasteur, Hopkins/UF, Imperial, Duke), while the other half were stochastic simulation models (Exeter/Oxford, Notre Dame, UF, UWA). The epidemiological and vaccine parameters used by the four deterministic models were based on empirical literature estimates and on estimates derived from fitting to published aggregated data from the active and hospital phases of the Dengvaxia Phase III trials [8,9]. The Sanofi Pasteur model was fitted to active phase data alone but also made use of unpublished disaggregated data from the trials [24] and hence was able to estimate serotype-specific differences in efficacy and epidemiological differences between countries. After initial fitting, parameters of the Sanofi model were later tuned to better represent the results of the first year of long-term follow-up. The stochastic models used a combination of parameters from the literature, from the deterministic model fits, and/or from fitting to dengue transmission in sites other than the trial sites.

**Table 1:** Comparison of models contributing to the exercise and overview of main differences. Further details are provided in S1 Appendix Chapter 1 and Tables C and D.

Group	Model type	Model fitted to Phase III trials?	Uncertainties represented	Seasonality	Demography
Sanofi Pasteur	Deterministic, nonspatial	Yes	Parameters and initial conditions	Biting rate, vector density	Brazil-like
Hopkins/UF		Yes	Initial conditions	Transmission	Brazil-like
Imperial		Yes	Parameters and initial conditions	Transmission	Brazil-like
Duke		Yes	Parameters	No	Brazil-like
UF	Stochastic, spatial	No	Parameters, stochasticity, and initial conditions	Vector density, incubation period	Mexico
UWA		No	Stochasticity	Vector density, incubation period	Thailand
Notre Dame		No	Parameters and stochasticity	No	Peru
Exeter/Oxford		No	Stochasticity	Vector density	Generic (67.5 y mean lifespan)

The models differed in assumptions and parameters relating to the natural history and ecology of dengue in both humans and mosquitoes as well as in the host demographics assumed. In addition, the models varied in what sources of uncertainty they incorporate (Table 1 and S1 Appendix).

### Model of Vaccine Action

**Fig 1:**
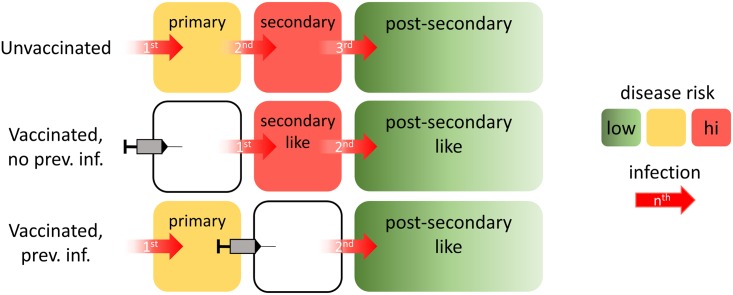
Illustration of the assumed vaccine mode of action. Without vaccination (top row), an individual will (by definition) experience a primary infection first, followed by a secondary infection, and then postsecondary infections. For vaccinees seronegative at the time of vaccination (middle row), their first natural infection behaves immunologically as a second natural infection would. Subsequent infections would immunologically behave as postsecondary infections. For vaccinees seropositive at the time of vaccination, any subsequent infection would immunologically behave as a postsecondary infection. The bottom row depicts such a case, in which the vaccinated individual has previously experienced only a single dengue infection. Because all postsecondary infections are assumed to have the same risk of disease, vaccination of individuals who have already had two infections would not modulate the risk of disease for subsequent infections. Boxes are color-coded according to the level of disease risk thought to be associated with primary, secondary, and postsecondary infections. The specific risks of developing dengue disease differ by modelling group (S1 Appendix Tables B and C).

The groups assumed a consistent model of vaccine action: vaccination mimics a silent (asymptomatic) natural infection in providing short-lived heterologous protection (all except UWA, who assumed a serotype-specific probability of life-long protection in seropositives) and modifying the probabilities of symptomatic and severe disease outcomes in subsequent natural infections in the same manner that a natural infection would have done (Fig 1). This vaccine model provides a parsimonious explanation of the active phase and long-term follow-up results reported in the Phase III trials [27,29]. Details of the specific assumptions for all models are provided in S1 Appendix Chapter 1 and Table A.

### Trial Simulation

To simulate the Southeast Asian Phase III trial (CYD14), all models represented a single setting in which the vaccine was delivered in a three-dose schedule at low coverage to children between 2 and 14 y old. The simulation was run for 3 y postvaccination, representing the 2-y active monitoring phase and the first year of passive follow-up. The models assume that all cases of symptomatic dengue and hospitalised dengue in trial participants were reported during that period. Predictions were compared with aggregated trial results on (i) age-stratified seropositivity at time of vaccine receipt, (ii/iii) age- and serostatus-stratified attack rates for virologically confirmed dengue in both the vaccine and the control arms during the active phase, and (iv) age-stratified hospitalisation rates for dengue in both the vaccine and the control arms during the first year of the long-term follow-up. Some models used the simulations to fit their models to the observed data (aggregated over all countries), and the remaining models used those parameter estimates for their simulations (Compare Table 1 and S1 Appendix Table B). With the exception of the transmission intensity, parameter values obtained from fitting or calibrating the trial simulations to the trial data were used in predicting the long-term impact of routine vaccination. No ethics approval was required for the secondary analyses of these published data.

### Routine Vaccination and Transmission Scenarios

The default policy considered was routine vaccination of 9-y-old children in a three-dose schedule, in which doses were administered 6 mo apart. Perfect compliance of the three-dose schedule was assumed with a coverage of 80%. Alternative strategies examined 50% coverage, addition of a three-dose catch-up campaign in 10- to 17-y-olds at 80% vaccine coverage in the first year of vaccine introduction, and alternative ages of vaccine administration between 10 and 18 y.

These vaccination strategies were modelled for a variety of levels of dengue endemicity (i.e., transmission intensity). Transmission intensity was characterised by the average proportion of 9-y-olds who are seropositive prior to vaccine introduction (labelled SP9), and models were run for the following transmission scenarios: 10% (very low transmission intensity), 30% (low), 50% (moderate), 70% (high), and 90% (very high). For comparison, the baseline seropositivity rate in children 9 to 12 y old in the trial settings in Asia and Latin America was between 48% (Mexico) and 91% (Colombia) [30]. Other studies of dengue seroprevalence (i.e., the proportion of individuals who have experienced at least one infection with any dengue serotype) in endemic areas have found values of SP9 in the range of 40% to 81% [31–34].

For each combination of vaccination strategy and transmission intensity, we examined the impact of vaccination on infections, symptomatic dengue, hospitalised dengue, and deaths over a 10- and 30-y period after vaccine introduction. Case definitions for symptomatic dengue and hospitalised dengue were chosen in accordance with the Phase III trials.

The Phase III trials did not report any dengue-associated deaths and were not powered to look at the impact of vaccination on mortality. We therefore assumed that vaccine efficacy against death was the same as that against hospitalised dengue disease. Specifically, each model assumed that a fixed proportion of hospitalised cases will die. The probability of death was assumed to be in the range of 0.03% to 0.09% for symptomatic dengue cases (S1 Appendix Table B), based on a review of surveillance data and published studies from Latin American countries [1,35–38].

### Health Economic Evaluation

The cost-effectiveness of vaccine introduction was evaluated following WHO guidelines [39]. Health effects were measured in terms of disability-adjusted life years (DALYs). A time horizon of 30 y was used, but benefits from averted mortality were accrued over the entire lifetime of the individual. Costs and health effects were discounted at 3% per year. Treatment costs were selected to broadly reflect an upper-middle-income Latin American country and were inflated to 2014 $USD. A public health care payer perspective was used (i.e., we did not include societal costs such as payment for private sector care in the analyses). Sensitivity analyses were conducted using (i) a societal perspective (using the friction cost approach to calculate the economic consequences of premature mortality [40]), (ii) no discounting for health effects, or (iii) alternative cost parameters broadly reflecting a lower middle-income Southeast Asian country. Additionally, parameters governing costs and DALYs associated with fatal and nonfatal dengue cases, respectively, varied by ±50%.

As both vaccine procurement and delivery costs are unknown, outcomes are presented in terms of the threshold cost per fully vaccinated person for vaccination to be cost-effective (i.e., the maximum amount that can be paid to fully vaccinate one person while remaining cost-effective). DALYs averted were monetized using values ranging from US$0 to US$10,000 that allowed comparison to other interventions but with a rate of US$2,000 for each DALY averted in the base case (S1 Appendix Table C).

Unit costs for treatment and DALYs incurred as a result of dengue were estimated from the literature (see Table 2; details in S1 Appendix Table C). The results broadly indicate the regional cost-effectiveness of vaccination on average and should not be interpreted as the cost-effectiveness in any particular country, which would require a more focused study that evaluated local costs and disease burden.

**Table 2:** Overview of assumed DALYs and costs used in health economic analysis, together with references that were used to estimate (after rounding) these values.

	Middle income Latin American–like setting	Lower middle income Southeast Asian–like setting
**DALYs incurred**		
Symptomatic dengue	0.006 [41]	0.006 [41]
Severe dengue	0.02 [41]	0.02 [41]
**Costs from public payer perspective ($USD)**		
Symptomatic case	60 [42]	20 [10,43]
Hospitalised case	200 [9,42]	400 [8,43]
**Costs from societal perspective ($USD)**		
Symptomatic case	200 [8,9]	40 [8,9]
Hospitalised case	500 [8,9]	500 [8,9]
Fatal case	11,000	3,000

## Results

### Trial Simulation

**Fig 2:**
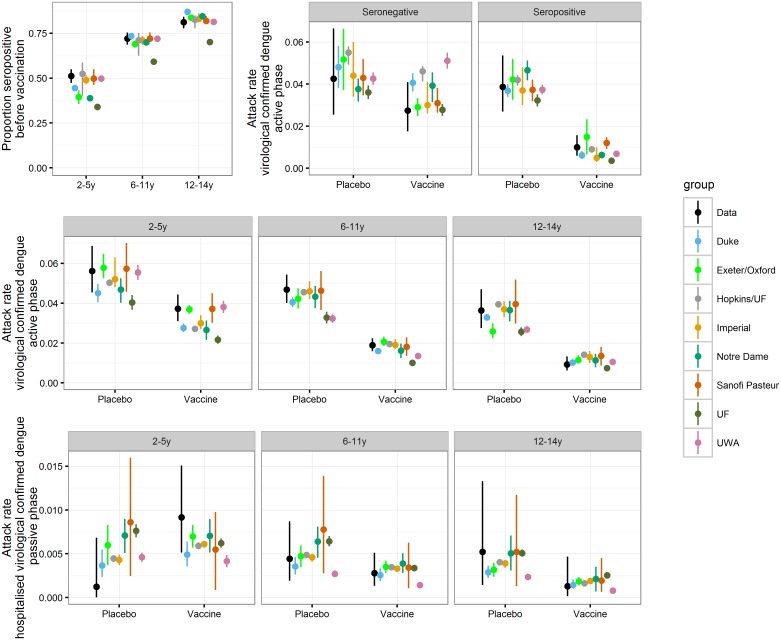
Comparison of aggregated CYD14 Phase III and long-term follow-up trial results with model predictions. For the data, (black) dots report mean estimates and error bars report 95% binomial confidence intervals. For model predictions, dots report mean estimates and error bars report the 95% range of simulations.

All models matched the aggregate data well, with modelled point estimates generally overlapping the confidence intervals of the observed data (Fig 2). Most models captured qualitatively the increased risk for hospitalisation in vaccinated 2- to 5-y-olds during the first year of the long-term follow-up; however, they predict it to be substantially lower than the observed point estimate (Fig 2 and S1 Appendix Figure M). Conversely, all models predict the vaccine efficacy for the 12–14 y age group to be lower than the observed point estimate.

### Transmission Dynamics in the Absence of Vaccination

In the absence of vaccination—and despite differences in realisations of underlying demographics—all models generated similar age distributions of disease incidence (S1 Appendix Figure A) and seroprevalence (S1 Appendix Figure B). In settings with very low transmission intensity (SP9 = 10%), the burden of DENV disease was fairly evenly distributed among age groups in the absence of vaccination. At higher transmission intensities, disease burden shifted towards childhood, with 35% to 70% of all dengue hospitalisations predicted to occur in children younger than 9 y.

### Population Impact of Routine Vaccination of 9-Y-Olds

**Fig 3:**
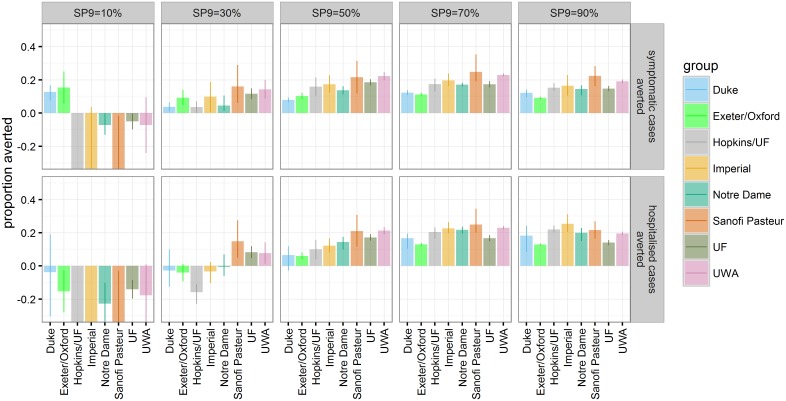
The proportion of symptomatic and hospitalised DENV cases (rows) averted within 30 y after vaccine introduction in the reference scenario for the range of transmission intensities (columns). The bars represent the mean and the error bars represent the 95% range over multiple simulations for each model (values are provided in S1 Appendix Table D).

In high-transmission settings (SP9 ≥ 70%), models consistently predicted that routine vaccination of 9-y-olds at 80% vaccine coverage would reduce symptomatic and hospitalised dengue incidence, with the magnitude of mean reduction varying by model between 13% and 25% (range of all simulations: 8%–34%) over the first 30 y of the policy (Fig 3 and S1 Appendix Table D). In settings with very low transmission intensity (SP9 around 10%), most models predicted an increase in symptomatic DENV cases, while two models (Duke and Exeter/Oxford) predicted a decrease. These two models predicted a beneficial effect of vaccination on reducing symptomatic infections because routine vaccination of 9-y-olds at 80% coverage in a setting with low prevalence resulted in an appreciable herd immunity effect, thereby reducing dengue circulation and the overall number of infections and cases (S1 Appendix Figure G). All models predicted an increase in DENV hospitalisations at this very low transmission intensity following vaccine introduction. This included the Duke and Exeter/Oxford models, for which the reduction in the overall number of infections was not sufficiently large to offset the increased severity of “secondary-like” breakthrough dengue infections in vaccine recipients. In our assumed vaccine mode of action, vaccination immunologically primes seronegative recipients, causing their first natural dengue infection to have the higher severity associated with secondary infection in unvaccinated individuals. This leads to overall increases in the incidence of hospitalised infection in settings where transmission is low enough for a substantial proportion of the population to not be expected to experience secondary infection in the absence of vaccination.

In settings with low to moderate transmission intensity (SP9 = 30%–50%), predictions were more variable between models. At SP9 = 30%, models differed over whether the impact of vaccination on hospitalised DENV cases was predicted to be detrimental or beneficial. The four models that showed the most favourable vaccine effect among 2- to 5-y-olds in first year of the long-term follow-up of the trials (S1 Appendix Figure M) predicted a positive impact of vaccination at age nine on hospitalisations in this setting, while the other four predicted a negative impact. Similarly, the latter four models predicted a lower impact of vaccination at age nine on hospitalisations at SP9 = 50%, although all models predicted a decrease in hospitalisation risk following vaccination in this setting.

In settings with SP9 ≥ 50%, the cumulative number of averted DENV cases was predicted to accrue almost linearly over time (S1 Appendix Figure E). This is due to the fact that, in most models, the main impact from vaccination is due to direct protection of vaccines rather than indirect effects on transmission (S1 Appendix Figure G)—a result of the only transient protection against dengue infection provided by the vaccine. In contrast, any potential increase in either symptomatic cases or hospitalisations following vaccination in the SP9 ≤ 30% settings was only visible after a honeymoon period of about 10 y (S1 Appendix Figure E).

Vaccination was found to impact only moderately on dengue transmission and the proportion of seropositive children at time of vaccination in subsequent years (S1 Appendix Figure C), with less than a 22% reduction in the hospitalisation rate of children too young to be vaccinated in all scenarios (S1 Appendix Figure G). Transient protection against infection following vaccination had limited effect on transmission, except in settings with very low transmission intensity in some models (see models by Duke and Exeter/Oxford). However, those models which assumed higher transmissibility of secondary infections (Sanofi Pasteur, Hopkins/UF, Imperial UF; S1 Appendix Table B) predicted that in moderate- to high-transmission settings, where many of such secondary infections with high transmissibility are averted, up to 20% of hospitalisations of children too young to be vaccinated could be averted.

### Individual-Level Impact of Routine Vaccination of 9-Y-Olds

**Fig 4:**
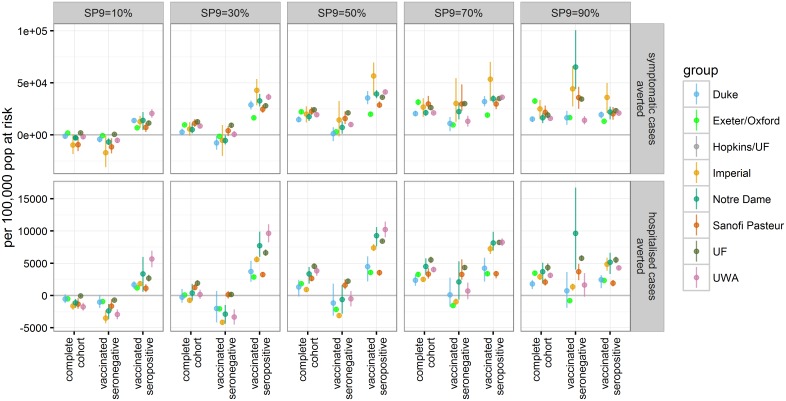
The number of symptomatic and hospitalised DENV cases averted per 100,000 population in the first vaccinated cohort within 30 y after vaccination. The effects of vaccination are shown for three groups: the complete first vaccine-eligible cohort, those individuals who were seronegative at time of vaccination, and those who were seropositive at time of vaccination (values are provided in S1 Appendix Table E).

Since the trial results suggest that serostatus (i.e., whether an individual has experienced at least one dengue infection with any serotype in the past) is a key driver of vaccine efficacy, we further examined the impact of vaccination and its relationship to an individual’s serostatus. For this, the first vaccinated cohort was followed over 30 y postvaccination. In each transmission setting, vaccination substantially reduced the risk of symptomatic or hospitalised disease in recipients who were seropositive at the time of vaccination. Conversely, seronegative individuals were predicted to be at increased risk for hospitalisation in settings with low to moderate dengue endemicity (SP9 ≤ 30) (Fig 4 and S1 Appendix Table E), with the increased risk persisting into settings with moderate and high endemicity for some models.

### Effect of Varying Vaccination Coverage

Reducing vaccine coverage from 80% to 50% reduced the impact of vaccination proportionately, both for instances in which the vaccine is predicted to be beneficial and in which it is predicted to increase the risk for symptomatic dengue or hospitalisation. As a consequence, the proportion of cases averted per vaccine dose given is similar for 50% and 80% uptake (S1 Appendix Figure H).

### Impact of Catch-Up Campaigns

Adding a one-off, three-dose catch-up campaign among 10- to 17-y-olds at 80% coverage in the first year of introduction to the default policy of routine vaccination of 9-y-olds increased the impact of vaccination. The impact of such a one-off campaign was most visible in the first few years. The transient protection against DENV infection induced by the catch-up campaign in a large proportion of school age children led to a temporary reduction in transmission, followed by a rebound in incidence in some of the models, dependent on the transmission setting and breadth of the catch-up campaign (S1 Appendix Figure J). However, over the long term, most models predicted that a one-off catch-up campaign prevented a similar number of DENV hospitalisations per dose of vaccine delivered as the baseline routine vaccination strategy (S1 Appendix Figure I).

### Effect of Changing Age for Routine Vaccination

**Fig 5:**
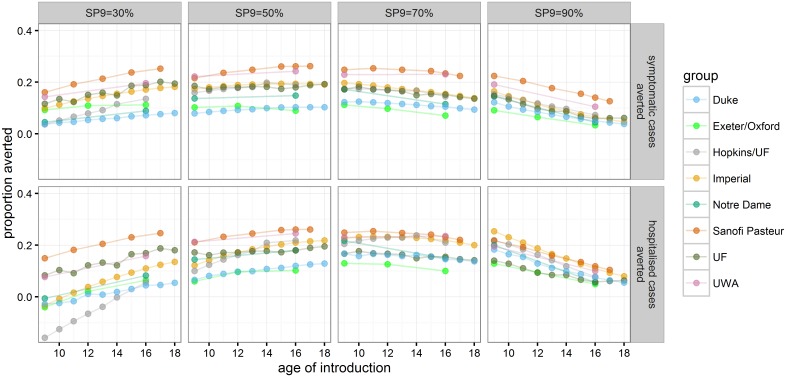
The proportion of symptomatic and hospitalised DENV cases averted in the 30 y after vaccine introduction. Each point represents a mean across model realization at a given age of vaccine introduction (values are provided in S1 Appendix Table F).

Alternative ages for routine vaccination were explored in the range of 9–18 y (Fig 5 and S1 Appendix Table F). In the highest transmission setting (SP9 = 90%), vaccination at age 9 gave the largest impact on symptomatic and hospitalised cases, leading to a range of mean reduction in hospitalised cases of 13%–25% (range of all simulations: 8%–31%) compared to a 5%–12% (range of all simulations: 3%–16%) reduction if 16-y-olds were vaccinated. Varying the age of routine vaccination between 9 y and 18 y in settings with SP9 = 50% or 70% showed that the maximal difference in the proportion of hospitalised cases averted is smaller than 5% for most models. Although the predicted optimal age of vaccination varied, in most models this was 10 y–14 y for SP9 = 70% and at the maximum age of evaluation for SP9 = 50%. In low-transmission settings (SP9 = 30%), all models predicted that the highest impact was achieved when vaccinating at the highest age examined. In settings with SP9 ≥ 30%, all models predicted a beneficial population impact on both symptomatic and hospitalised cases if vaccination is targeted at individuals 14 y of age or older (S1 Appendix Table F).

### Cost-Effectiveness

**Fig 6:**
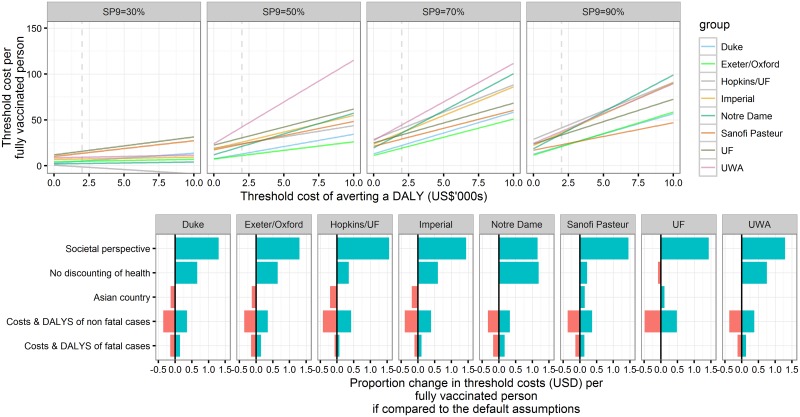
Threshold cost per fully vaccinated person for the base case and sensitivity analyses. Upper panel: Threshold costs per fully vaccinated person in reference to thresholds of the cost of averting a DALY. Cost and health outcomes are calculated for 30 y after the introduction of Dengvaxia to 9-y-olds with 80% coverage and without a catch-up campaign. The public health care provider’s perspective is taken, and both health and costs are discounted at 3%. Lower panel: Sensitivity analyses on the threshold costs per fully vaccinated person in a highly endemic setting (SP9 = 70%), assuming the threshold costs of averting a DALY are US$2,000 (values are provided in S1 Appendix Table G).

Using base case economic assumptions (3% discounting of costs and DALYs, threshold cost per DALY averted of US$2,000, public health care provider perspective and middle-income Latin American country–like costs), the range of mean threshold cost per vaccinated person across models in the 50%–90% seroprevalence setting was predicted to be US$11–US$44 (range of all simulations: US$8–US$52) (Fig 6 and S1 Appendix Table G). Generally, vaccination was most cost-effective for SP9 = 70%. In low-transmission-intensity settings (SP9 = 30%), all models predicted a threshold cost per fully vaccinated person below US$16.

For most models, the threshold cost per fully vaccinated person changed by less than 50% if leaving health effects undiscounted, using Southeast Asian costs, or varying costs and DALYs associated with fatal or nonfatal cases. The range of predicted mean threshold costs across models rose to US$26–US$115 (range of all simulations: US$18–US$156) and US$28–US$104 (range of all simulations: US$21–US$117) per fully vaccinated person in settings with SP9 ≥ 50% if the threshold cost per DALY averted under base case economic assumptions was increased to US$10,000 or a societal perspective was adopted (S1 Appendix Fig K).

In line with the model predictions for health impacts, cost-effectiveness was maintained (or even improved) for later ages of vaccination, except in the 90% seroprevalence setting. The incremental cost-effectiveness of a one-off catch-up policy was found to be similar to that of vaccinating 9-y-olds routinely in moderate-transmission settings but lower in the highest (SP9 = 90%) transmission intensity setting.

## Discussion

The results from this comparative modelling study indicate that Dengvaxia has the potential to reduce the burden of dengue disease in moderate- to high-transmission-intensity settings (SP9 ≥ 50%). This range of transmission intensity includes most sites selected for the Phase III trials of Dengvaxia. The greatest impact of vaccination was predicted for high-transmission-intensity settings (SP9 ≥ 70%), where routine vaccination of 9-y-olds at 80% coverage was predicted to reduce DENV-related hospitalisations by between 13% to 25% over the first 30 y following vaccination introduction. However, in settings with low transmission intensity (SP9 ≤ 30%), we predict that vaccination could increase the incidence of dengue-related hospitalisations. The per-dose impact of vaccination was similar when reduced vaccine coverage and the impact of a catch-up campaign were examined. Targeting older children could increase the net benefit of vaccination in settings with low and moderate transmission intensity. Overall, vaccination was predicted to be potentially cost-effective at a threshold of US$2,000 per DALY across all models in moderate- to high-transmission settings if the costs of vaccinating an individual could be kept well below approximately US$50 (from a provider perspective) or US$100 (from a societal perspective).

These findings are based on the assumption that the vaccine mimics a natural and clinically silent infection. While only longer-term follow-up will show if these assumptions hold, at present they present a parsimonious explanation of the observed results from Phase IIb and III trials. Under these assumptions, in low-transmission settings, where most individuals are typically dengue naïve at the age of 9 y, any breakthrough natural infection following vaccination would result in a secondary-like infection outcome, including an elevated risk of disease and hospitalization. In those settings, vaccination at an older age may mitigate some of these effects by allowing more time for children to experience their first natural infection prior to vaccination. In settings with higher transmission intensities, most children will have had exposure to dengue prior to vaccination and hence vaccination would bypass the dengue infection associated with the highest disease risk (as these individuals’ next infection would act as a postsecondary infection). Furthermore, in these highly endemic settings, even children who are seronegative at time of vaccination are highly likely to experience at least two natural infections in their life, so the net effect of vaccination is just to lower the age at which they experience a “secondary-like” infection rather than to increase the overall incidence of such infections. However, in children who have already experienced two or more infections at time of vaccination, the vaccine-preventable burden of disease is small because postsecondary infections only rarely result in severe clinical manifestations. Hence, in settings with extremely high transmission intensity, the impact of vaccination of 9-y-olds is likely to be smaller than in the scenario of highest endemicity that we explored.

While model consensus on these qualitative features is strong, there is some discrepancy on the level of transmission intensity where Dengvaxia transitions from a detrimental to a beneficial impact on dengue disease. Those models that include a better efficacy of Dengvaxia against hospitalisation of 2- to 5-y-old vaccine recipients in the first year of the long-term follow-up predict a beneficial net impact of a routine vaccination programme at lower transmission intensity.

All models predicted that routine use of Dengvaxia in populations who are largely dengue naïve may increase the burden of dengue disease. This indicates that national decisions on the implementation of vaccination programmes for dengue will need to identify regions of high transmission intensity and the appropriate population to target for vaccination. We chose the seroprevalence at nine years of age as a proxy measure for transmission intensity. Considering that such data are not available at a sufficient subnational resolution in most endemic countries, other proxy measures, including existing syndromic surveillance, may be able to be used. However, the translation between those proxies and seroprevalence will need further evaluation.

With the implementation of new vaccines, safety monitoring should be in place to detect adverse events which are too infrequent to be detectable in trials. The results reported here suggest that a potential safety concern of Dengvaxia is an increased risk of dengue-related hospitalisation for vaccinees who were dengue naïve at time of vaccination, which may only be observable years after vaccination, particularly in low-transmission settings. Routine safety monitoring systems are insufficient to capture this unique risk, and carefully designed Phase IV studies that can account for interseason variability of dengue incidence are needed to assess whether vaccine rollout leads to increases in the incidence of symptomatic or hospitalised dengue in parts of the population.

At a threshold cost per DALY averted of US$2,000, most of the benefit of vaccination in all the models came from averting health care costs rather than DALYs. However, at a threshold cost per DALY averted of US$10,000, the value of DALYs averted became more important than health care costs averted. The number of deaths prevented by vaccination is particularly uncertain because dengue case fatality risks vary widely by setting; vaccine impact on dengue mortality is not directly informed from trials, and case fatality risks tend to be higher in young children, an age group likely to benefit little from Dengvaxia. Hence, the uncertainty around estimates with a high threshold cost per DALY averted is greater than when the threshold cost per DALY averted is at US$2,000.

All models adopted biological assumptions regarding vaccine action that the SAGE Working Group on Dengue Vaccines and Vaccination agreed best reflected current understanding. However, current limitations of the available trial data mean uncertainties remain regarding the level of protection provided against disease versus infection and the rate at which vaccine-induced protection declines. In particular, the assumption that vaccination acts in a similar way to natural infection is consistent with the phase III trial results but cannot be directly validated given the lack of information on the impact on asymptomatic infections or baseline serostatus in the vast majority of trial participants. Similarly, there is no information about the impact of a postvaccination infection on the immune state of a seronegative vaccinee; all models made the plausible, but optimistic, assumption that such individuals would have immunity comparable to that of someone who had experienced two natural infections [29], but there are no data available to compare this and other plausible scenarios.

Analyses of trial data stratified by both age and serostatus have indicated that serostatus is a more important driver of vaccine efficacy than age. Nevertheless, we cannot rule out there might be intrinsic variation in vaccine efficacy with age, independent of serostatus; if efficacy is higher in older recipients, vaccine impacts are likely to be larger than presented here (or less detrimental in low-seroprevalence settings). Vaccine efficacy may also vary by serotype (independent of serostatus); only the Sanofi Pasteur model was able to explore this in detail, as trial data disaggregated by both country and serotype (needed to fit to serotype-specific efficacies) are not currently publically available.

Important limitations also derive from the data used to calibrate the models against. The long-term follow-up has not yet been completed, is based on passive surveillance, and was not designed to assess vaccine efficacy. As a consequence, conclusions drawn from it rely on a limited number of dengue cases. Also, the trials only provide data from settings with a seroprevalence of 9- to 12-y-olds of at least 48%.

Furthermore, underlying uncertainties about dengue epidemiology also affected our predictions. Perhaps the most pertinent to the predictions of vaccine impact shown is the relationship between symptoms and infectiousness. Whereas many of the models assumed that symptomatically infected individuals were substantially more infectious than those who were asymptomatic, several others assumed that symptoms did not alter infectiousness. Empirical evidence for a relationship between symptoms and infectiousness is mixed [44,45]. If infectiousness is not correlated with disease, the positive impact of vaccination in the respective models in the high-transmission settings is likely to be slightly lower than the impact predicted here by these models, while the predicted negative impact in low-transmission settings could equally be reduced.

The results from the second year of long-term follow-up have recently become available. In particular, they show that in year 2 of the long-term follow-up in the Southeast Asian trial, children vaccinated at 6–11 y old were at higher risk for hospitalisation because of dengue than their unvaccinated controls (although this finding was not seen in the Latin American trial). Including this additional data did not significantly alter our parameter estimates and, hence, our prediction of the impact of routine vaccination (see S1 Appendix Figure N). However, none of the models were able to reproduce the elevated risks in 6- to 11-y-old vaccinees. A possible explanation is waning of protection in seropositive recipients; all models presented here assumed such protection to be lifelong. If protection in seropositive recipients does wane, our predictions of vaccine impact presented here may be overoptimistic. However, additional data from year 3 of the long-term follow-up are needed to test this and other hypotheses on vaccine action.

### Conclusion

Informed by the results of this work, the WHO SAGE committee recommended countries consider introduction of Dengvaxia only in national or subnational settings with high endemicity—as defined by seroprevalence of approximately 70% or more in the targeted age group—and recommended against its use in age groups with seroprevalence <50% [13,15]. Decisions at the country level for vaccine introduction may be informed by this work but should also take into account local dengue epidemiology, predicted impact, and cost-effectiveness, with country-specific inputs as well as local priorities, affordability, and capacity for introduction and postlicensure monitoring. To complement a rigorous review of clinical trial data, mathematical modelling provides an important opportunity to predict the impact of vaccination programs that is otherwise difficult to anticipate using clinical trial data when vaccine performance is variable by host or setting characteristics.

## Supporting Information

S1 Appendix Additional information on individual models, additional results, and tabulated outcomes. (DOCX)

## Abbreviations

ADE: antibody-dependent enhancement
CMDVI: comparative modelling of dengue vaccine public health impact
DALY: disability-adjusted life year
DENV: dengue virus
SAGE: Strategic Advisory Group of Experts
