# Supplementary material for: The Long-Term Safety, Public Health Impact, and Cost-Effectiveness of Routine Vaccination with a Recombinant, Live-Attenuated Dengue Vaccine (Dengvaxia): A Model Comparison Study
Source: PLoS Med. 2016 Nov 29;13(11):e1002181. doi: 10.1371/journal.pmed.1002181 (PMC5127514; doi:10.1371/journal.pmed.1002181)
Supplement: S1 Appendix — (DOCX) [file pmed.1002181.s001.docx]

**Appendix to**

**“The long term safety, public health impact, and cost effectiveness of a routine vaccination programme against dengue: a model comparison study”**

Stefan Flasche^1^*, Mark Jit^1^*^+^, Isabel Rodríguez-Barraquer ^2^*, Laurent Coudeville^3^*, Mario Recker^4^*, Katia Koelle^5^*, George Milne^6^*, Tom Hladish^7^*, Alex Perkins^8^*, Derek Cummings^2,7^, Ilaria Dorigatti^9^, Daniel Laydon^9^, Guido España^8^, Joel Kelso^6^, Ira Longini^7^, Jose Lourenco^10^, Carl A.B. Pearson^7^, Robert C. Reiner^11^, Luis Mier-y-Terán-Romero^2^, Kirsten Vannice^12^, Neil Ferguson^9^

## Detailed model descriptions

### Duke

The Duke dengue transmission model is a 4-serotype deterministic ordinary-differential equation epidemiological model that was originally presented in Nagao & Koelle (2008) [1]. In this publication, three distinct models were considered, differing from one another in the type of transient cross-protection brought about by a natural dengue infection. Here, we adopted the clinical cross-protection assumption, which assumes that infection with one serotype confers transient heterologous protection from clinical disease, not infection. Because there is no protection from infection, an individual who is challenged by a heterologous serotype during a period of cross-protection will seroconvert to the heterologous serotype, such that a subsequent exposure to this serotype can no longer result in infection (or disease). Although an individual can become infected and seroconvert while in the cross-protected state, infection is assumed to result in very low viremia, such that the individual does not become infectious. We therefore assume that there is no potential for onward transmission from individuals in cross-protected immune states.

The model we use assumes that all infected individuals, besides the one who are in cross-protected immune states, transmit similarly. It also assumes that susceptibility to infection (given no previous homologous infection) is independent of immune status, that is, the number of previous heterologous infections an individual has experienced. We assume a duration of infection of 9 days, a period of clinical cross-protection of 2 years following a natural dengue infection, no seasonality, and a low immigration rate to stabilize the epidemiological dynamics but not appreciably alter attack rates. This immigration rate varies by SP setting, with SP10% assuming an immigration rate of 1x10^-5^ infected individuals per serotype per year, SP30% assuming an immigration rate of 1x10^-4^ infection individuals per serotype per year, and the remaining higher transmission intensity settings each assuming an immigration rate of 1x10^-3^ infected individuals per serotype per year. The basic reproduction numbers corresponding to these transmission intensity settings were *R*_0_ = 1.12 (SP10%), 1.40 (SP30%), 1.80 (SP50%), 2.675 (SP70%), and 5.00 (SP90%). Demographic (birth/death) rates were chosen to reproduce the current age structure of Brazil. In line with the other dengue transmission models, we assume that vaccination acts like a silent natural infection. In our case, this means that it induces a transient period of clinical cross-protection. We use an 8-month period of vaccine-induced clinical cross-protection (rather than a 2-year period of clinical cross-protection induced by a natural dengue infection). This period was chosen by simulating the model under cross-protection periods ranging from 6 months to 2 years, and selecting the period with the highest likelihood value as determined by fitting to the CYD14 and CYD15 phase III trial data.

We estimated the progression rates of our model by fitting simultaneously to both the CYD14 and CYD15 phase III data, including the active phase results and the first year of the long-term follow-up. Specifically, we used Bayesian Markov Chain Monte Carlo to estimate the probabilities with which primary, secondary, and post-secondary dengue infections progress to symptomatic disease (defined as VCD), and the proportions with which VCD cases are hospitalised. These estimated parameters are provided under the Duke column in Table B. We assume that 0.078% of VCD dengue cases result in death.

Uncertainty in the outcomes of the simulated vaccine scenarios only reflects uncertainty in the progression rate parameters of the dengue transmission model. We sample 100 parameter sets from the posterior distributions we arrive at through fitting the CYD14 and CYD15 trial data, and plot the 95% range of outcome values, discarding the lowest and highest 2.5% of values.

### Exeter/Oxford

This agent-based model describes the transmission dynamics of four co-circulating dengue serotypes (DENV1-4) within a spatially-explicit meta-population setting, first presented in [2]. The spatial structure is realised through a regular grid of subpopulations, each consisting of a fixed number of humans and seasonally regulated number of mosquitoes. Both human and mosquito death rates are age-dependent and governed by a continuous Weibull distribution with average life-expectancies of 67.5 years and 20.2 days for humans and mosquitoes, respectively. Human individuals are considered to be susceptible, exposed (intrinsic incubation period= 2 days), infectious (duration= 4 days) or recovered with respect to each serotype, allowing up to four consecutive infections; differential infection outcomes were not explicitly modelled. Only the susceptible, exposed (extrinsic incubation period= 7 days) and infectious states of adult mosquitoes are considered.

Transmission events between humans and mosquitoes are probabilistic and predominantly within their own and directly adjacent communities, with a small probability of long-distance transmission. We assumed transmission-dependent human→vector and vector→human transmission probabilities, which offered greater flexibility for fitting the model to different transmission settings without having to change the general model structure. Due to the possibility of stochastic extinction, especially when considering low transmission settings, external virus introduction is considered at a fixed rate of 0.2 per day with a serotype chosen randomly (equating to 0.05 / serotype / day).

Apart from disallowing co-infections, no serotype immune interactions in the form of temporary cross-immunity or ADE are considered, and no distinctions are made regarding the length and/or transmissibility of primary, secondary or subsequent infections.

Parameter deviation from original model:

| Parameter | this model | original model |
| --- | --- | --- |
| Human population size | *N*_h_=3.6E6 | *N*_h_=1E6 |
| Vectors per human host | *M*=0.95-2 | *M*=0.7-1.2 |
| Weibull parameters | *a_h_*=2E-4, *b_h_*=10 | *a_h_*=5.5E-3, *b_h_*=5.5 |
| Extrinsic incubation period | 1/*δ*_v_=7 days | 1/*δ*_v_=6 days |
| Daily mosquito biting rate | *a*_v_=0.55 | *a*_v_=0.6 |
| Transmission probability (*h*→*v*) | *ε_hv_*= 0.28 (SP9=10%); 0.38 (SP9=30%); 0.435 (SP9=50%); 0.52 (SP9=70%); 0.565 (SP9=90%) | *ε_hv_*=0.5 |
| Transmission probability (*v*→*h*) | *ε_vh_*= 0.28 (SP9=10%); 0.28 (SP9=30%); 0.335 (SP9=50%); 0.41 (SP9=70%); 0.565 (SP9=90%) | *ε_vh_*=0.5 |

In line with the CMDVI agreed action of Dengvaxia®, vaccination was administered in three doses six months apart and acts as a silent infection that moves individuals up the infection ladder. It also provides temporary heterologous protection against infection, where protective efficacy wanes exponentially over time (from 100% at the point of the last vaccine dose to 0% with a half-life of 1 year) regardless of the immune status of the individual.

All transition rates (infection→VCD, VCD→hospitalisation) were determined by fitting the model outcome (within a transmission setting at 70% seroprevalence in 9 year olds) to the CYD14 vaccine trial data using a Bayesian Markov Chain Monte Carlo method, resulting in the following age- and exposure-dependent progression rates:

|  |  | 1-8 years | 9-14 years | 15-17 years | | 18+ years |
| --- | --- | --- | --- | --- | --- | --- |
| infection → VCD | primary infection | 0.439 | 0.439 | | 0.439 | 0.439 |
|  | secondary infection | 0.540 | 0.370 | | 0.324 | 0.308 |
|  | post-secondary infection | 0.209 | 0.144 | | 0.126 | 0.119 |
| infection → hospitalisation | primary infection | 0.011 | 0.011 | | 0.011 | 0.011 |
|  | secondary infection | 0.107 | 0.073 | | 0.064 | 0.061 |
|  | post-secondary infection | 0.019 | 0.013 | | 0.012 | 0.011 |

### University of Florida

Our model differs from the previously published version [3] primarily in terms of parameter assumptions.  For this work, we assume several values which differ from those we fit or assumed for the Yucatan forecasts:

| Parameter | Original fit mean / fixed value | Assumed value for CMDVI | Discussion in [3] |
| --- | --- | --- | --- |
| Primary infection pathogenicity relative risk | Age-stratified | 0.75 | SI 1.3 |
| Base (secondary DENV1 infection) pathogenicity | 0.69 | 0.60 |  |
| Post-secondary pathogenicity relative risk | 0.1 | 0.167 |  |
| Primary severe proportion | 0.012 | 0.06 |  |
| Secondary severe proportion | 0.025 | 0.13 |  |
| Post-secondary severe proportion | 0.005 | 0.033 |  |
| External exposure rate | 0.18 per day | 0.1 per day | SI 1.4 |

We also use only the subset population for Merida and corresponding fit parameters (see SI 5.4 in [3]), similar to approach for initial fitting there, but also used for the CMDVI forecasts.

Our original model reports mild and severe disease outcomes, which differs from the desired outcomes for the CMDVI work: symptomatic cases, hospitalized cases, and deaths.  To obtain the CMDVI outcomes from our model output, we apply the following transformation to obtain hospitalized cases:

$$hosp=severe+0.11*mild$$

Deaths are a simple linear transformation of hospitalized cases (see Table C).

To obtain the desired seroprevalence levels in 9 year olds, we use a force-of-infection multiplier, which we apply to the mosquito population size.  That parameter was fit by iteratively guessing multipliers, performing regression on the multiplier-SP9 surface, and then re-guessing multipliers.  Relative to our base parameter fits, we obtained the following multipliers:

| SP9 | Force of infection multiplier |
| --- | --- |
| 10% | 0.622 |
| 30% | 0.852 |
| 50% | 1.09 |
| 70% | 1.47 |
| 90% | 2.29 |

Finally, our implementation of the vaccine model is identical to our waning model (see SI 4.4 in [3]), but with a 1 year half-life and an initial efficacy of 100%.

### Imperial

We developed a deterministic, age-stratified (1 year age groups up to age 20, then 10 year age groups to age 100) compartmental four-serotype dengue transmission model [4]. The model represents the dynamics of the female mosquito population; mosquitoes become infected upon feeding on an infectious human, then remain infectious for the rest of their life. Humans are born susceptible and can experience four infections by heterologous serotypes. Infection gives permanent immunity against the homologous serotype and exponentially decaying (mean of 1 year) cross-protection against infection with heterologous serotypes. The chance that infection is symptomatic varies by how many infections an individual has experienced in their life, with these parameters estimated by fitting the model to the phase 3 trial data. We assume that symptomatic infections are twice as infectious as asymptomatic infections. Vector competency and population sizes are assumed to vary seasonally.

The main effect of vaccination is to act as a ‘silent’ natural infection, boosting immunity (and the risk of disease) in seronegative recipients to that experienced by unvaccinated individuals who have only experienced one natural infection (Figure 1). Similarly, recipients who have already experienced one natural infection prior to vaccination have their immunity boosted to that of unvaccinated individuals who have experienced two natural infections. Vaccination was also assumed to give waning heterologous protection against infection, the duration of which was estimated by fitting the model to the phase 3 trial data to be 9 months (95%CI: 3-11 months). The initial efficacy of this transient protection was also estimated, separately for seronegative and seropositive recipients, but was not significantly different from 100% for seronegative recipients, and not significantly different from 0 for seropositive recipients.

The model was fitted to both phase 3 trials simultaneously. The model likelihood had 4 components: (a) clinical attack rates of virologically confirmed dengue disease in years 1 and 2 of the active phase of the trials, by age group; (b) active phase clinical attack rates for the immunogencity subset of the trials, by baseline serostatus; (c) proportion of the immunogenicity subsets who were seronegative prior to receiving their first vaccine or placebo dose, by age group; (d) hospitalisation rates for virologically confirmed dengue disease in the first year of the long-term follow-up phase of the trials, by age group. Three parameters were fitted as trial-specific: the starting time of the trial relative to the start time of the simulation (the model produces chaotic epidemic dynamics, so fitting the start time of the trial is akin to fitting initial conditions, albeit under the constrain that the model dynamics are in pseudo-equilibrium), the basic reproduction number of dengue in the trial population (*R*_0_), and the probability of hospitalisation given symptoms. Six parameters were estimated as being the same for both trials: the probabilities of symptomatic infection for primary, secondary and post-secondary infections, the initial vaccine efficacy against infection in seronegative and seropositive recipients, and the mean duration of that protection against infection.

When computing forward projections of the potential impact of vaccination strategies, we generated 70 model realisations for each combination of transmission intensity and age at vaccination. Each realisation used a randomly selected start time for vaccination (between 150 and 250 years after the start of the simulation, to allow the dynamics to equilibrate) and a random sample from the joint posterior density of the estimated parameters. Each realisation was run with and without vaccination. We recorded the proportion of symptomatic dengue disease and hospitalized dengue case incidence averted by vaccination each month over 50 years.

### Hopkins/UF

We developed a deterministic, four-serotype, age-stratified compartmental dengue transmission model that includes explicit vector dynamics as well as cross-protection between dengue serotypes. It is a four-serotype extension of our previously published model [5]. Briefly, humans are assumed to be born susceptible and can undergo up to four infections by heterologous serotypes.  Upon infection, only a fraction of incident infections progress to symptomatic disease and the rest remain asymptomatic. After each infection, there is a period of cross protection in which individuals can’t be infected by heterologous serotypes. Following the cross-protected state (mean duration of 1 year), individuals enter a state in which they are susceptible to all of the serotypes they have not encountered. Our mosquito population consists of all females; individuals are born susceptible and become infected upon feeding on humans infected with said serotype. We do not account for a period of virus incubation in the vectors and once infected they remain infectious for the rest of their life span.

Our model assumes that the progression rates to symptomatic disease depend on the infection number. We estimated these progression rates by fitting our model to the CYD14 and CYD15 trial data (see below). Our model also assumes that symptomatic individuals transmit twice as much as asymptomatic individuals, but performed sensitivity analyses varying this assumption. Based on epidemiological studies conducted in Thailand, we assume some heterogeneity in transmissibility between serotypes [6]. In particular, we assume that DENV-2 is the most transmissible, and DENV-1, 3, and 4 are 0.85, 0.81 and 0.82 as transmissible, respectively. We assumed a stable population structure and chose demographic (birth/death) rates to reproduce the current age structure of Brazil.

Given the uncertainty regarding the mode of action of the vaccine, we fit several competing models to the CYD 14 and CYD 15 data, as well as to the long term follow-up data. In all cases, we estimated several vaccine parameters (vaccine efficacy in seropositive and seronegative individuals and duration of protection) as well as natural history parameters (disease progression rates in primary-quaternary infections and hospitalization rate). These parameter estimates are reported in Table B. Models were fit in the Bayesian MCMC framework.

Consistent with other models presented here, the best fitting model assumes that vaccination acts like a silent natural infection. As such, it leads to short-term cross protection against infection (mean duration of 1.1 years) and moves individuals down the infection line. Thus, for example, primary infections in individuals that are vaccinated while fully susceptible progress to symptomatic disease at the rate of secondary infections in unvaccinated individuals.

For each vaccination scenario, we ran 50 simulations varying the timing of vaccine introduction. Uncertainty in simulated scenarios of vaccine roll-out represents variation due to the timing of introduction.

### Notre Dame

This model is a stochastic, individual-based model with four dengue serotypes circulating in a population that is configured to resemble the city of Iquitos, Peru. Aspects of the model that are well informed by data from Iquitos include the geographic layout of individual houses and other buildings, population-level demographic composition, household demographic composition, human movement patterns, and biting heterogeneity within houses. Aspects of the model that are currently not informed by data from Iquitos include mosquito densities, population immunity, and patterns of dengue importation from surrounding areas that presumably seed and replenish transmission within the city. The overall result of these strengths and limitations is that the model is highly realistic in its description of the spatial distribution and mixing patterns of people but represents many other factors in a flexible way determined by a limited number of free parameters. The model is described in full detail by Perkins et al. [7].

A parameter sweep was conducted in the original analysis of the model to assess the relationships between SP9 after 40 years of transmission and four free parameters: mosquito emergence rate, mosquito death rate, infectiousness of mosquitoes to people, and the force of infection due to importation of dengue from elsewhere. Variation in SP9 across the parameter sweep was best explained by a combination of variation in the force of importation parameter and vectorial capacity, a standard metric of transmission intensity and a composite of the latter three of the model’s free parameters and some of its fixed parameters. To obtain values of the free parameters that would yield SP9 values consistent with the 10-90% scenarios, we first chose a range of values of the force of importation parameter (1.0x10^-5^, 3.25x10^-5^, 5.5x10^-5^, 7.75x10^-5^, 1.0x10^-4^) and the mosquito infectiousness parameter (0.2, 0.4, 0.6, 0.8, 1) that represented a broad range of values explored in the parameter sweep. We furthermore adopted a single value of daily mosquito mortality of 0.11 across all scenarios. Finally, we selected values of mosquito emergence rate of 0.20, 0.25, 0.29, 0.35, and 0.46 that, in combination with the other parameters, resulted in SP9 values consistent with the 10-90% SP9 scenarios.

The vaccine was implemented consistent with harmonized assumptions, as were progression rates to VCD, hospitalization, and death. Efforts were made to use this model to fit parameters describing how the vaccine works—i.e., degree and half-life of temporary infection blocking—but no values of those parameters performing better than the harmonized values of 100% and 1 year were found. This does not preclude the possibility that better-fitting values of these parameters exist, but if they do exist they were not identifiable based on a crude parameter sweep approach to seeking better-fitting values.

All realizations of the model were simulated for 40 years prior to vaccination to allow for the build-up of population immunity and another 30 years following the introduction of the vaccine. Each realization of the model involving vaccination was paired with an otherwise identical realization of the model without vaccination. Each pair of simulations was identical in terms of two distinct random number seeds: one that pertained to random events related to demography, movement, biting, and importation of infections from outside the simulated population, and another that pertained to infection, disease, and vaccination outcomes. This resulted in pairs of simulations that were directly comparable and thus isolated the impacts of vaccination from stochastic noise to the greatest extent possible (although not completely). For each transmission and vaccination scenario, 100 replicates were performed and the average and interquartile range across the set of 100 replicates were reported for comparison with other models’ outputs.

### Sanofi Pasteur

This model is an age-structured, host-vector compartmental model accounting for the transmission dynamics of the four dengue serotypes. Vaccination was assumed to act as a silent infection but, in contrast with other models, its parameters were not estimated from publicly available summary statistics but from detailed, country-specific information collected during the active surveillance phase of Dengvavia® efficacy studies [8]. As a consequence, in contrast with other models, vaccine efficacy accounts for observed differences by serotype, higher efficacy against hospitalizations and increase in efficacy with doses for seronegative subjects. Point estimates for initial efficacy ranges from 13% (Dengue 2 - 1 dose for seronegative subjects) to 93% (Dengue 4, Hospitalised cases for seropositives). The relative risk of developing a symptomatic case or a severe case requiring hospitalization upon primary, secondary and post-secondary infection were also adjusted to best represent country-specific information.

Vaccine efficacy was assumed to provide partial protection to all vaccinees (leaky vaccine). For the results reported here, the annual waning rates of vaccine protection were 100% for seronegative subjects and 15% for seropositive subjects. Based on an analysis of immunogenicity data, vaccine efficacy against asymptomatic infections was considered to be 50% of the efficacy against ambulatory cases in the base case. This model also differs from most models regarding the evolution of symptomaticity with age. The comparison between reported surveillance data and model outcomes led us to consider an increased symptomaticy upon infection during childhood.

For all transmission intensity considered here, we used Brazilian demographic information and reported average and 95% confidence interval based on 100 simulations. For each of these simulations we drew values from the posterior distribution of the estimated vaccine efficacy parameters. We also considered different starting points for the introduction of the vaccine (with a 10 year reference period) and used a uniform distribution for the relative efficacy against asymptomatic infections (0–100% of the efficacy against ambulatory cases).

### UWA

The University of Western Australia (UWA) dengue model is a development of one produced previously for the city of Cairns in tropical Australia, where dengue epidemics occur almost every year [9]. In Cairns the *Aedes aegypti* population naturally enters a state of stasis in winter due to low minimum temperatures halting the development cycle of this dengue vector. The mosquito population starts to grow in spring when the weather warms and rainfall increases. Dengue transmission is thus not endemic and the lack of adult vectors in winter breaks ongoing transmission. Arrival of dengue infectious individuals in the spring restarts virus transmission, often leading to significant numbers of cases, with rigorous vector control being instigated to mitigate case numbers. Infectious individuals are seeded into the model and randomly located into specific households.

The model developed for this study is “Thai-like”; the *Aedes aegypti* population fluctuates according to the season in Thailand, with the population at a lower level in January and peaking during the wet season (April to October). This generates an annual peak in dengue transmission consistent with incidence data for central Thailand, i.e. in the region of Bangkok. The Thai vector population dynamics model is simplified from the Cairns model, omitting the immature (egg, larva and pupa) life stages and models only adult female mosquitos. The simulated human population of approximately 74,000 (average over a projected 30 year period) has the same household size, age structure and age-specific birth and death rates found in central Thailand as reported by the National Statistical Office Thailand (2010). Population size and ages predicted in the future are consistent with World Bank projections.

The Thai model used here, and the related Cairns model, are spatially-explicit allowing geographical variation of vector and human populations to be captured. These individual-based simulation models utilise census data to represent the make-up of households, and other data sources to estimate human movement between households, schools, workplaces and the wider community. Vector population dynamics follows standard techniques with temperature (impacting mosquito activity and reproduction rate) and rainfall (impacting availability of breeding sites) the main drivers in population development. The model includes a number of stochastic processes (e.g. contact of mosquito and human; biting events resulting in infection; vaccination outcomes) and thus multiple simulation runs (24 in fact) are required with averages taken to generate infection data over a 30 year time period. The reported 95% confidence intervals quantify the uncertainty in outcomes due to stochastic variation between different simulation runs.

All 4 dengue serotypes are included. Following an infection from a particular serotype, an individual will remain immune to that serotype for life. From the time at which an individual recovers from a given serotype, he/she will be immune to infection from other serotypes for 6 months (cross protection). There is no simultaneous co-infection; multiple serial infections can occur. The simulated average annual dengue incidence rate for a baseline no-vaccination scenario was calibrated to be consistent with the observed Thai average annual incidence rate, assuming the above and that dengue cases are under-reported by a factor of ~4, which is consistent with estimates [10]. The R_0_ measured from initial simulations was 3.3 (compare with 3.2 for Thailand in [5]) giving seropositivity among of 9 year olds (SP9) of 65%. For this study higher and lower SP9 settings, ranging from 10% to 90%, were created by adjusting the vector population density.

Vaccine effect is modelled as being “all-or-nothing”: successfully vaccinated individuals are fully protected from infection after the final vaccine dose, while unsuccessful vaccination offers no protection i.e. vaccination does not provide additional protection against symptomatic dengue or hospitalisation in the case of breakthrough infections. Successful versus unsuccessful vaccination is determined stochastically for each strain for each recipient, with a probability based on virus strain and sero-status. Vaccine half-life is assumed to be 1 year for seronegative individuals and life-long for seropositive individuals. In other respects vaccine effect is modelled as described in the Methods section.

The relatively small population modelled required ongoing seeding of infectious individuals to replicate the natural movement into and out of the modelled community. Much of the detail found in both the Thai and Cairns models developed at the University of Western Australia is unnecessary for the purposes of modelling dengue vaccination, as in this study (e.g. spatial vector density heterogeneity and vector movement). This resulted in long simulation times running on the Western Australian Pawsey Centre high-performance computing facilities; however, such detail is necessary to also capture vector control interventions, a future use of the UWA models.

**Table A: Model specific descriptions of the vaccine mode of action**

| MODEL | |  | Sanofi Pasteur | Hopkins / UF | Imperial | Duke | UF | UWA | Notre Dame | Exeter / Oxford |
| --- | --- | --- | --- | --- | --- | --- | --- | --- | --- | --- |
| Natural infection | | |  |  |  |  |  |  |  |  |
|  | Transient protection | | Life-long homologous protection, 1-year heterologous protection | Life-long homologous protection against infection; 1-year heterologous protection | Life-long homologous protection against infection; 1-year heterologous protection | Life-long homologous protection against infection; 2 year heterologous protection, against symptom development and onward transmission. | Life-long homologous protection against infection, 2-year heterologous protection. | Life-long homologous protection against infection, 6-month heterologous protection. | Life-long homologous protection against infection, 2-year heterologous protection | Life-long homologous protection against infection; No heterologous protection |
|  | Immunopathogenic response | | Increasing the immune memory of past infections by one | | | | | | | |
| Vaccine in seronegative | | |  |  |  |  |  |  |  |  |
|  | Transient protection | | Imperfect, serotype-specific protection against infection (increasing with disease severity) of 35% for 1.5 years | Protection against infection with any of the 4 serotypes of 1.1 years | Protection against infection with any of the 4 serotypes of 7 months | Protection against symptom development and onward transmission with any of the 4 serotypes of 8 month. | Protection against infection with any of the 4 serotypes of 1 year | Serotype specific probability of 1 year protection with subsequent 1 year half-life. | Protection against infection with any of the 4 serotypes of 1 year | Protection against infection with any of the 4 serotypes of 1 year |
|  | Immunopathogenic response | | Same as natural infection | | | | | | | |
| Vaccine in seropositive | | |  |  |  |  |  |  |  |  |
|  | Transient protection | | Imperfect, serotype-specific protection against infection (increasing with disease severity) of 75% for 10 years | None | None | Same as in seronegative recipients | Same as in seronegative recipients | Serotype specific probability of life-long protection. | Same as in seronegative recipients | Same as in seronegative recipients |
|  | Immunopathogenic response | | Same as natural infection | | | | | | | |

Table B: Parameter assumptions on progression probabilities from asymptomatic infection through to death, on the variance in transmissibility with severity, and on variation from the baseline vaccine mode of action. Ranges indicate the 95% range of the posterior estimates.

| **MODEL** |  | **Sanofi Pasteur** | **Hopkins / UF** | **Imperial** | **Duke** | **UF** | **UWA** | **Notre Dame** | **Exeter / Oxford** |
| --- | --- | --- | --- | --- | --- | --- | --- | --- | --- |
| Factor by which transmissibility is increased in symptomatic infections | | 4 | 2 | 2 | 1 | 2x / 3x for mild / severe infections | 1 | 1 | 1 |
| Proportion of VCD among infections | primary infection | from 10% (<5 y) to 31% (>15 yrs) | 53% | 45%  (30%-70%) | 34.2%  (27.1% - 41.4%) | 45% | 30% | 30% | 32% |
|  | secondary infection | from 16% (<5 y) to 52% (> 15 yrs) | 100% | 85%  (55%-100%) | 71.4%  (64.4% - 78.6%) | 60% | 60% | 60% | 38% |
|  | tertiary infection | from 4% (<5 y) to 13% (> 15 yrs) | 23% | 11%  (6%-17%) | 13.6%  (9.8% - 17.8%) | 10% | 10% | 10% | 12% |
|  | quarternary infection | same as tertiary | 0% | same as tertiary | same as tertiary | same as tertiary | same as tertiary | same as tertiary | same as tertiary |
|  | age dependency? | yes | no | no | no | no | no | no | yes |
| Proportion of hospitalised cases among VCD | primary infection | ~4% of infections (~11% of cases) | 0% | 0.5% of infections (~1% of cases) | 5.3% of cases (0.2% - 15.1%) | all severe cases, ~10% mild cases | 11.1% | 11.1% | 3% |
|  | secondary infection | ~7% of infections (~15% of cases) | 14% | 10% of infections (~12% of cases) | 11.2% of cases (7.2% - 15.4%) | ibid | 20.9% | 20.9% | 11% |
|  | tertiary infection | ~1% of infections (~7% of cases) | 0% | 0.1% of infections (~0.9% of cases) | 4.0% of cases (0.02% - 14.4%) | ibid | 5.2% | 5.2% | 2% |
|  | quarternary infection | ~1% of infections (~7% of cases) | 0% | 0.1% of infections (~0.9% of cases) | Assumed same as tertiary | ibid | 5.2% | 5.2% | 2% |
|  | age dependency? | yes | no | no | no | no | no | no | yes |
| proportion of deaths among VCD | | ~0.05%  (0.5% of hospitalised) | ~ 0.03%  (0.4% of hospitalised) | ~0.05%  (0.5% of severe cases) | 0.078% | 0.078% of hospitalised | 0.078% | 0.078% | ~0.084% (0.4% of hospitalised) |

## Pre-vaccination baseline comparison

We assessed the age distribution of symptomatic and hospitalised cases in the models. In settings with low transmission intensity the risk for dengue disease is almost equally distributed among all age groups (Figure A). With minor differences, the models agree that with increasing transmission intensity the burden of dengue disease becomes dominant in children less than 20 years old. Within the age group that was evaluated for potential introduction of routine vaccination in this work (children 9 to 18 years old), most models predict a gradual increase in seroprevalence with age. In settings with low, moderate, or high transmission intensity seroprevalence in children 18 years old was predicted to be about 25% higher than in 9 year olds (Figure B). The UWA model is highly stochastic in nature and while the trends of increasing seropositivity with age are similar to the other models they are partially mask by stochastic effects.


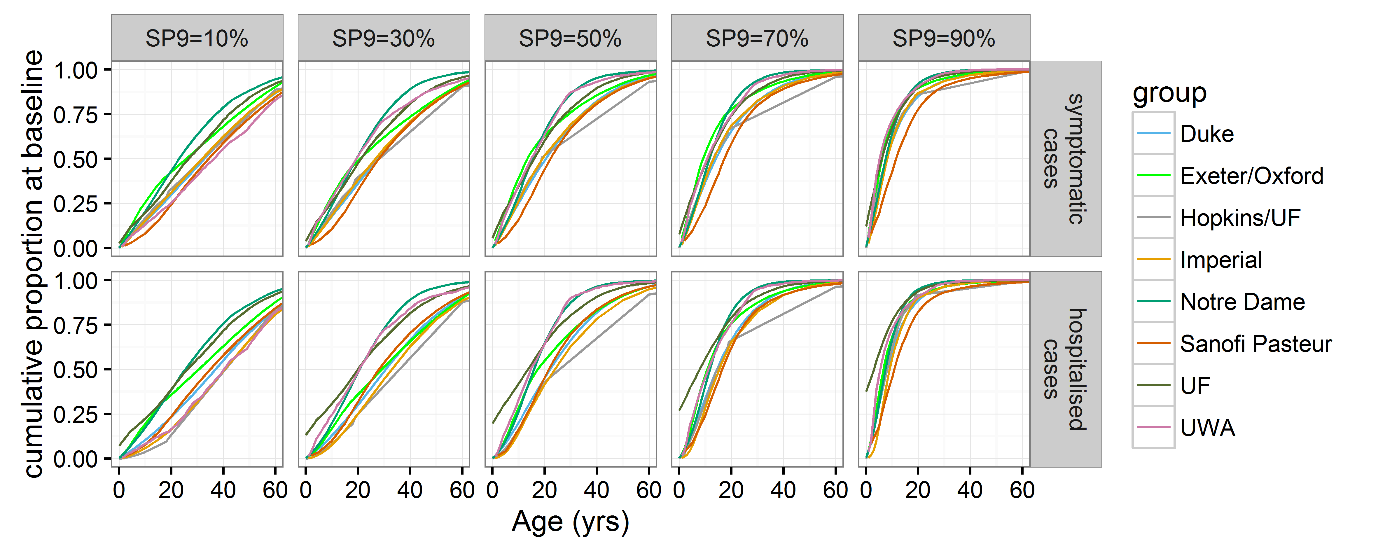


Figure A: The cumulative age distribution of symptomatic cases and hospitalised cases before the start of vaccination


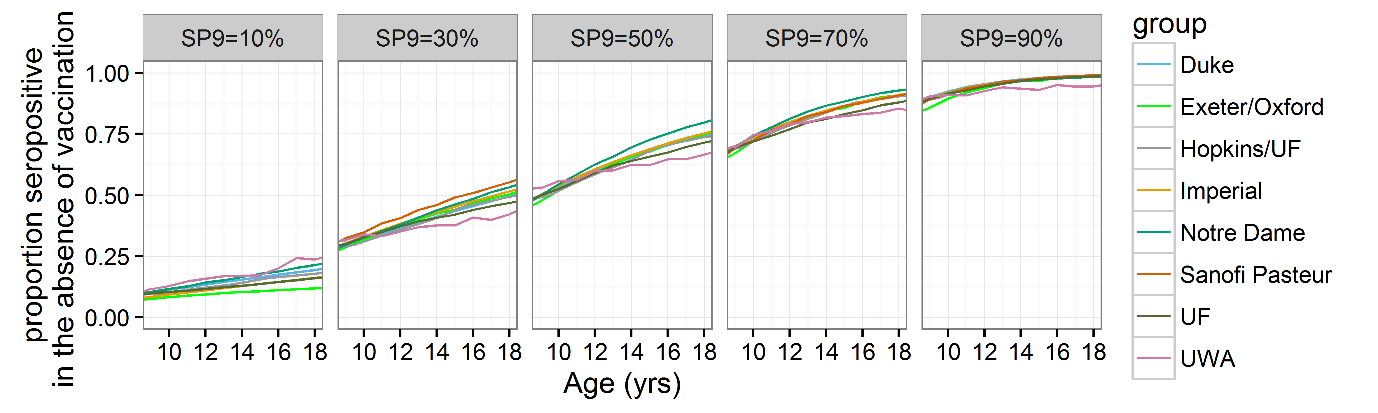


Figure B: The age-stratified proportion of seropositive individuals before the start of vaccination

## Additional post vaccination results

As we assumed only transient vaccine protection against dengue infection, in most models introduction of Dengvaxia to 9 year olds at 80% coverage did not substantially reduce transmission. In particular, those models which assumed that symptomatic cases are more infectious predict that in settings with low transmission intensity dengue circulation may be increased as a result of an increased probability of symptomatic infections in individuals who were seronegative at time of vaccination (Figure C). Similarly, the mean age of dengue infection stays mostly constant after vaccination. Only in the UWA model the mean age of infection increases slightly. This is caused by a small decrease in the rate of transmission due to the accumulation of individuals with long-term vaccine protection in this model (Figure D). The benefit of vaccination was generally predicted to accrue almost linearly over time in settings with at least moderate transmission intensity. In other settings most models predict relatively little impact during the 10 years after introduction of Dengvaxia® and (negative) effect to only accrue thereafter (Figure E and Figure F).


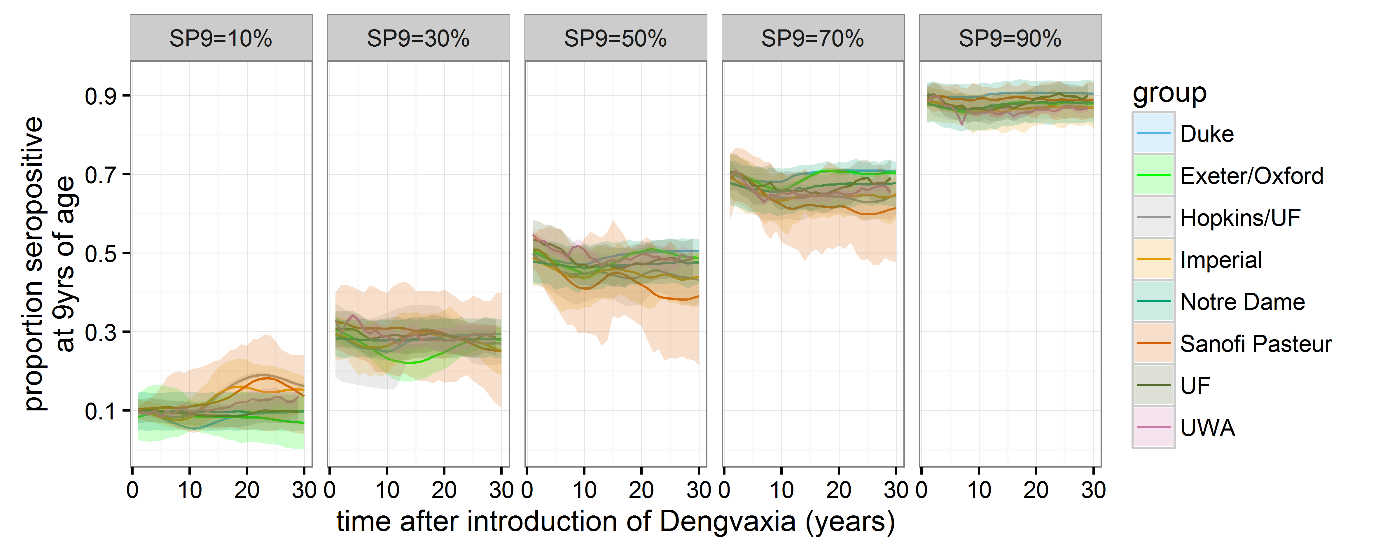


Figure C: The change in proportion of 9y old children that are seropositve at vaccination in the 30 years after the introduction of Dengvaxia®. Lines represent the mean and the shaded region of the respective colour the 95% range over multiple simulations


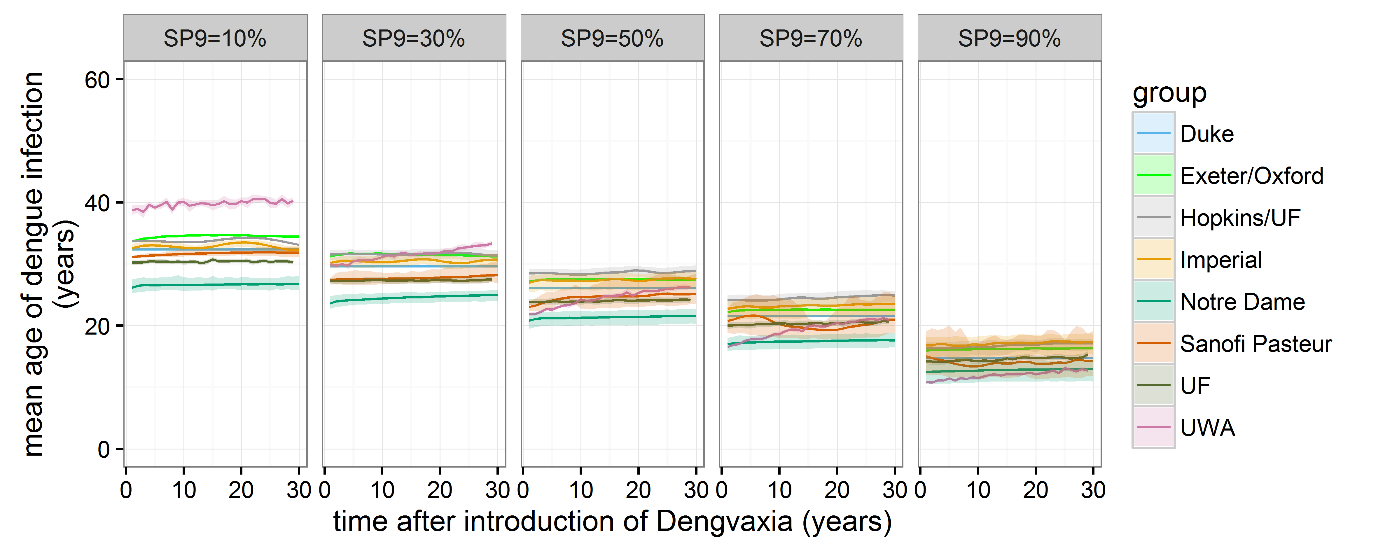


Figure D: The change in the mean age of infection in the 30 years after the introduction of Dengvaxia®. Lines represent the mean and the shaded region of the respective colour the 95% range over multiple simulations


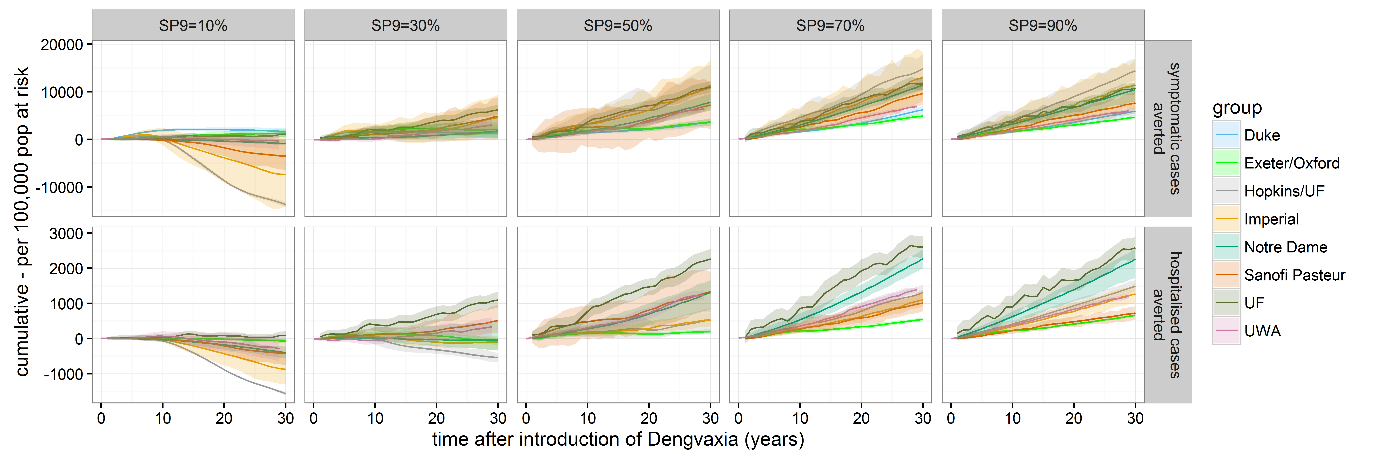


Figure E: Predicted cumulative number of symptomatic and hospitalised DENV cases averted per 100,000 population over the 30 years after the start of routine vaccination (reference scenario). Lines represent the mean and the shaded region of the respective colour the 95% range over multiple simulations.


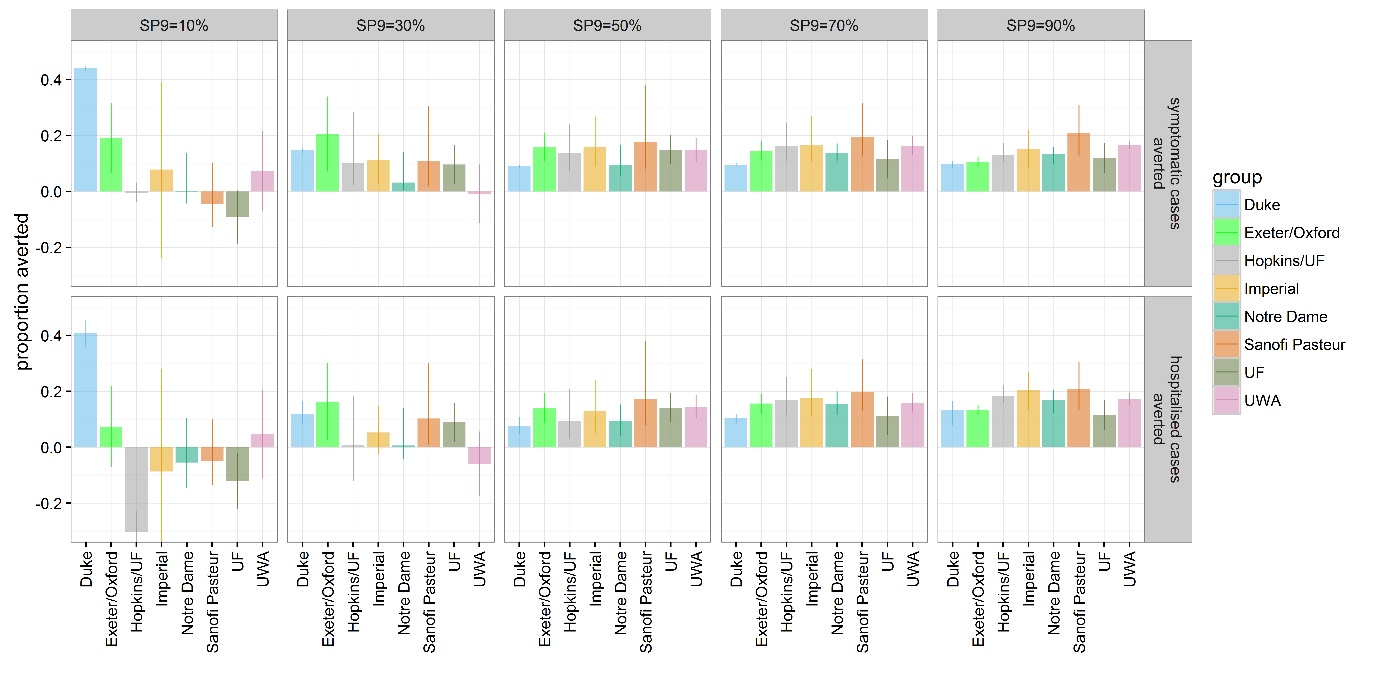


Figure F: The proportion of symptomatic and hospitalised DENV cases averted within 10 years after vaccine introduction in the reference scenario. The bars represent the mean and the error bars the 95% range over multiple simulations.


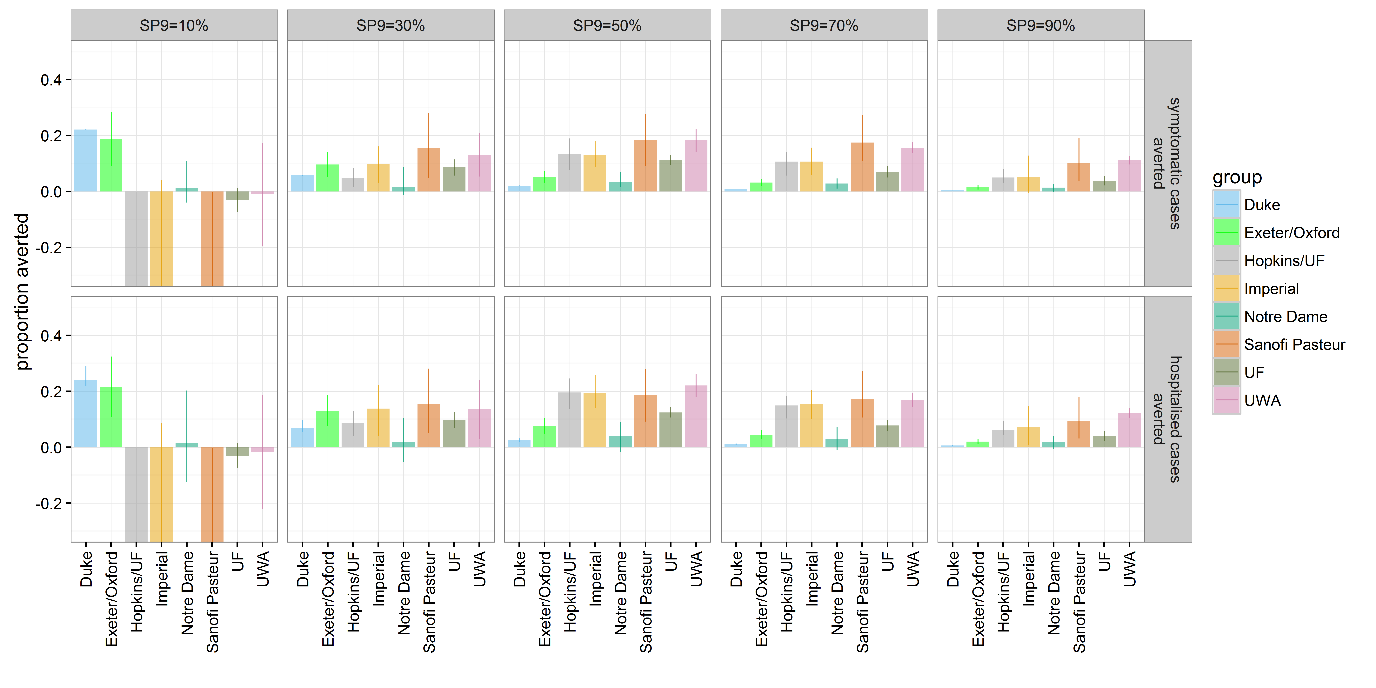


Figure G: The proportion of symptomatic and hospitalised DENV cases averted among unvaccinated children younger than 9 years within 30 years after vaccine introduction in the reference scenario. The bars represent the mean and the error bars the 95% range over multiple simulations.

Reducing vaccine uptake from 80% to 50% reduced the impact of vaccination proportionately. Since the majority of the predicted impact is gained from direct vaccine effects the proportion of cases averted per vaccine dose given is similar for 50% and 80% uptake (Figure H) and likely to scale accordingly for any other vaccine uptake. Similarly, adding a one-off 3-dose catch-up campaign among 10 to 17 year olds at 80% coverage in the year of vaccine introduction increased the impact of vaccination proportionally with the extra doses administered. The impact of such a one-off campaign was most visible in the first few years and hence was evaluated 10 years after the start of vaccination (Figure I). The transient protection against DENV infection induced in a large proportion of school age children by the catch-up campaign led to a small temporary reduction in transmission in some of the models (Figure J). Most models predicted that a one-off catch-up campaign prevented a similar number of DENV hospitalisations per dose of vaccine delivered as the baseline routine vaccination strategy.


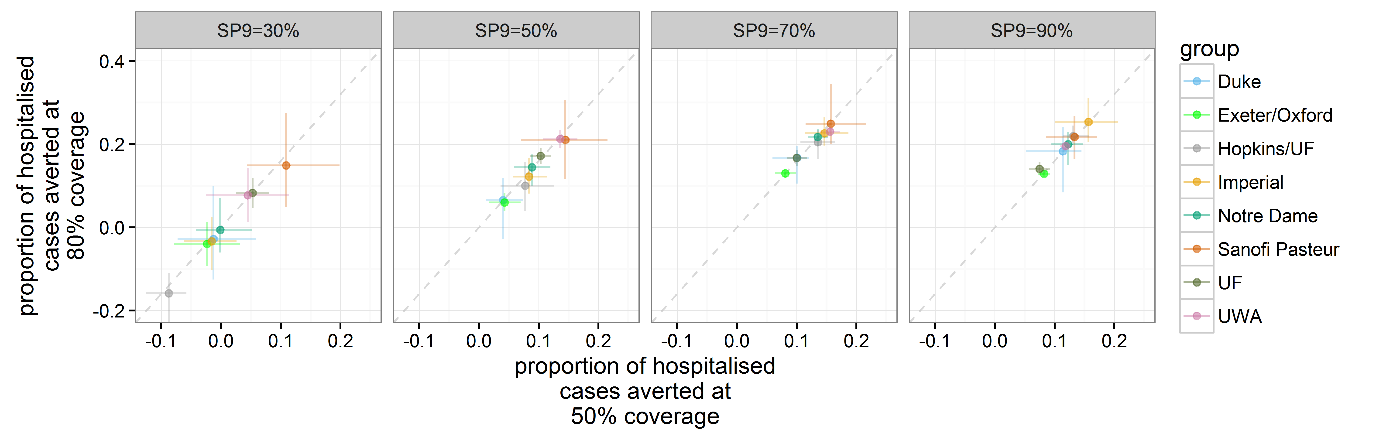


Figure H: The proportion of hospitalised DENV cases averted in the 30 years after vaccine introduction comparing vaccine coverage of 80% with vaccine coverage of 50%. The grey, dashed line represents those instances where both strategies prevent the same number of cases per dose of vaccine assuming that after 10 years the latter strategy has used 5/8 times less doses. SP9=10% scenario not shown for clarity (most models predicted an increase in long-term population risk of DENV hospitalisations for this scenario).


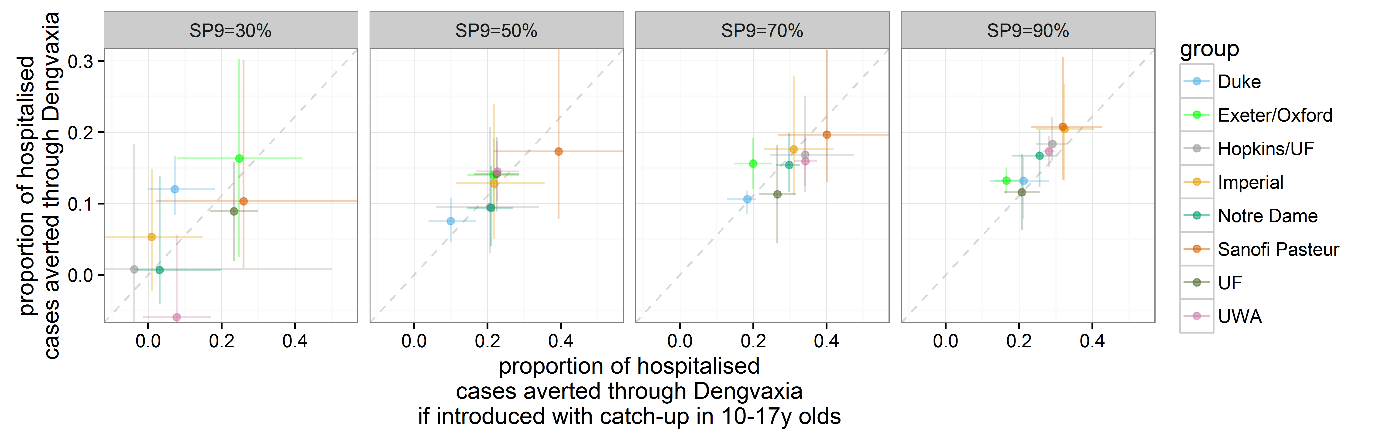


Figure I: The proportion of hospitalised DENV cases averted in the 10 years after vaccine introduction comparing introduction according to the reference strategy to introduction by adding a 3-dose catch-up campaign among 10 to 17 year olds. The grey, dashed line represents those instances where both strategies prevent the same number of cases per dose of vaccine assuming that after 10 years the latter strategy has used 18/10 times more doses. SP9=10% scenario not shown for clarity (most models predicted an increase in long-term population risk of DENV hospitalisations for this scenario).


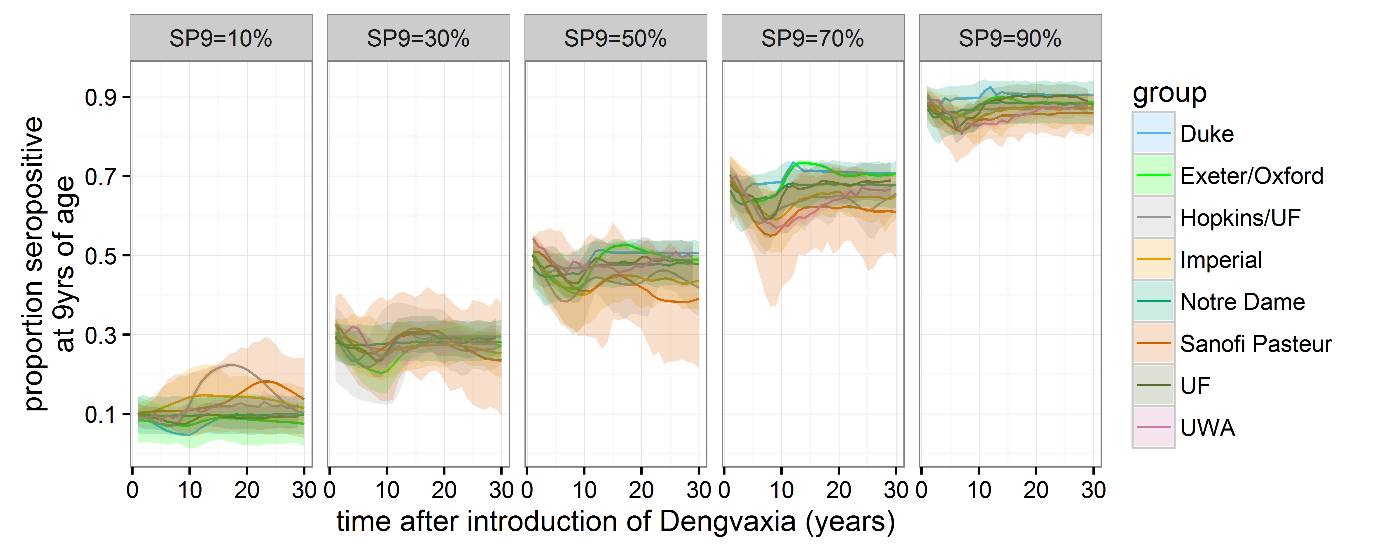


Figure J: The change in proportion of 9y old children that are seropositve at vaccination in the 30 years after the introduction of Dengvaxia® if introduced with a 3-dose catch-up campaign among 10-17 year olds. Lines represent the mean and the shaded region of the respective colour the 95% range over multiple simulations

## Parameter estimation for health economic evaluation

Direct and indirect unit costs of dengue treatment were estimated using data from Brazil as a representative of an upper middle income Latin American country. As a sensitivity analysis unit costs estimated in the Philippines are used, representing a lower middle income South-East Asian country. The use of these parameters should not facilitate interpretation of the cost-effectiveness analyses as being representative of dengue vaccination in Brazil or the Philippines. This would require a single-country economic evaluation using local epidemiological data. Final economic inputs were rounded to a single significant figure before being used.

*Ambulatory care.* The cost of public sector ambulatory care for dengue was estimated to be $64 in Brazil [11] and $124 in the Philippines [12]. In the Latin American like setting, we assumed that almost everyone with symptomatic dengue receives formal (hospital or ambulatory) care. In the placebo arm of the Dengvaxia trials, 43/389 or 11% of participants with virologically-confirmed dengue sought hospitalisation in the Latin American centres (CYD15) [13], so we assumed that 89% seek ambulatory care. By applying an incidence density of 6.7% in the intention-to-treat placebo arm of the Philippines trials of Dengvaxia to the 0-14 year old population in 2010 we estimate that around 1,000,000 cases of symptomatic dengue occurred in the Philippines [10]. One study based on expert elicitation suggests that around 300,000 ambulatory cases of dengue occur in the Philippines every year [12], so we assumed that 30% of people with symptomatic dengue seek ambulatory care. Hence the average cost per symptomatic dengue case was assumed to be $64 x 89% = $57 (round to $60) in Latin America and $124 x 30% = $37 (rounded to $40) in South-East Asia.

*Inpatient hospital care.* The cost of public sector hospital care for dengue was estimated to be $237 in Brazil [11] (rounded to $200) and $400 in the Philippines [12].

*Productivity.* The societal cost of lost productivity due to dengue was estimated as the daily gross domestic product per capita multiplied by the number of days of illness. We assumed that six days were lost for a non-hospitalised symptomatic case (based on the duration of symptoms for virologically-confirmed dengue in Dengvaxia trials [14,13]) and nine days for a hospitalised case (based on the duration of symptoms for severe dengue in Dengvaxia trials [14,13]). This translates to costs of $184 (rounded to $200) and $45 (rounded to $40) per ambulatory case in Brazil and the Philippines respectively, and $276 (rounded to $300) and $68 (rounded to $100) per hospitalised case. This was added to actual costs of care to get the overall cost to society. For fatal cases, we assumed that a year of productivity was lost, translating to $11,208 (rounded to $10,000) and $2,765 (rounded to $3,000) per death in Brazil and the Philippines respectively.

*DALYs incurred.* A disability weight of 0.545 was used, based on the weight for dengue haemorrhagic fever in the 2004 update to the Global Burden of Disease study [15]. Dengue and severe dengue cases were assumed to have symptoms lasting 4 and 14 days respectively.

To calculate the threshold cost per vaccinated person (maximum amount that could be paid to procure and deliver three doses of vaccine), DALYs averted by vaccination were converted into monetary values using a threshold cost per DALY averted. The threshold cost per DALY averted is the incremental cost to the health service or to society of saving an extra year of disability-free life. The use of ad hoc GDP per capita based thresholds for decision making has been criticised [16,17] and is now strongly discouraged by WHO. Instead, we take the approach of comparing the ICERs of other similar interventions (other new vaccines and dengue interventions) that may presumably be displaced by investment in dengue vaccination (see Table C). The ICERs for rotavirus, pneumococcal and human papillomavirus vaccination in comparable settings have been estimated to be in the range of $200 - $2,000 [18–20]. Case management of dengue has been estimated to have an ICER of around $200, while chemical and environmental vector control may cost around $2,000 - $14,000 (in 1993 USD, so likely even higher today) depending on the setting [21]. Hence we set a base case threshold of $2,000 but vary this up to $10,000.

However, up to now most international health economic evaluations have used a discount rate of 3% a year for both costs and health effects. Hence the ICERs of these interventions (and hence the threshold cost per DALY averted) would decrease if benefits were not discounted. To estimate the reduction, we calculated the ratio between the ICER of different vaccines in low and middle income country settings when health effects are discounted at 3% and 0% (in both cases, costs were discounted at the same rate as health effects because there are few vaccine studies that examine differential discounting). The ratio ranged from 0.22 (HPV vaccination in Brazil [18]) to 0.40 (rotavirus vaccination in Vietnam [22]) to 0.46 (pneumococcal conjugate vaccination in Kenya [23]). Hence we multiplied the threshold cost by 0.40 (to bring it to $800 - $4000) when using a discount rate of 0% for health effects.

Table C: Overview of costs and ICER of alternative intervention strategies to prevent dengue and of alternative vaccines.

| INtervention | Country | Cost per UNit | ICER | Currency | Reference | Perspective |
| --- | --- | --- | --- | --- | --- | --- |
| Hpv vaccination of 12-year olds | Brazil | 13.5 | 1,290 | 2011 USD | [18] | Provider |
| Hpv vaccination of 12-year olds | Philippines | 13.5 | 1,590 | 2011 USD | [18] | Provider |
| Hpv vaccination of 12-year olds | PAHO upper middle income countries min | 13.5 | 630 | 2011 USD | [18] | Provider |
| Hpv vaccination of 12-year olds | PAHO upper middle income countries max | 13.5 | 2,080 | 2011 USD | [18] | Provider |
| Hpv vaccination of 12-year olds | WPRO lower middle income countries min | 13.5 | 260 | 2011 USD | [18] | Provider |
| Hpv vaccination of 12-year olds | WPRO lower middle income countries max | 13.5 | 15,000 | 2011 USD | [18] | Provider |
| Infant pcv7 vaccination | Latin America & the Caribbean | 10 | 1,168 | 2005 USD | [19] | Provider |
| Infant pcv7 vaccination | Latin America & the Caribbean | 10 | 685 | 2005 USD | [19] | Societal |
| Infant rotavirus vaccination | Americas upper middle income countries | 7.5 | 506 | 2007 USD | [20] | Provider |
| Infant rotavirus vaccination | Western Pacific lower middle income countries | 7.5 | 439 | 2007 USD | [20] | Provider |
| Case management of dengue | Developed health system | 200 | 587 | 1993 USD | [21] | Provider |
| Vector chemically controlled (vs case management alone) | Developed health system | 0.46 | 6,568 | 1993 USD | [21] | Provider |
| Environmental vector control (vs case management alone) | Developed health system | 2.25 | 13,696 | 1993 USD | [21] | Provider |
| Vector chemically controlled (vs do nothing) | Undeveloped health system | 0.46 | 1,992 | 1993 USD | [21] | Provider |
| Environmental vector control (vs do nothing) | Undeveloped health system | 2.25 | 3,668 | 1993 USD | [21] | Provider |
| GDP per capita | Brazil | - | 11,000 | 2013 USD | World Bank | - |
| GDPdp per capita | Philippines | - | 2,765 | 2013 USD | World Bank | - |

## Additional health economic results

*Changes to the vaccination strategy.* In line with the model predictions for health impacts, cost-effectiveness is maintained (or even improved) for later ages of vaccination except in the 90% seroprevalence setting. The incremental cost-effectiveness of a one-off catch-up policy was found to be similar to that of vaccinating 9 year olds routinely in moderate transmission settings, but lower in the highest (SP9=90%) transmission intensity setting. This suggests that in moderate transmission intensity settings, the decision to implement a catch-up campaign should be driven by affordability considerations.

*Changes in economic assumptions.* Figure K shows the threshold cost per vaccinated person with changes in key economic assumptions around discounting, setting and perspective. Removing discounting of health effects has little effect in most models, since the threshold cost per DALY averted drops by 60% to reflect the better cost-effectiveness of alternative ways of spending on health under this scenario. Similarly, using a South-East Asian setting only alters parameters slightly and hence has little effect. However, using a societal perspective considerably raises cost savings, with threshold costs per vaccinated person increasing to $28-$104 for the base case threshold cost per DALY averted of $2,000 and reaching $43-$169 when the threshold cost per DALY averted is $10,000.


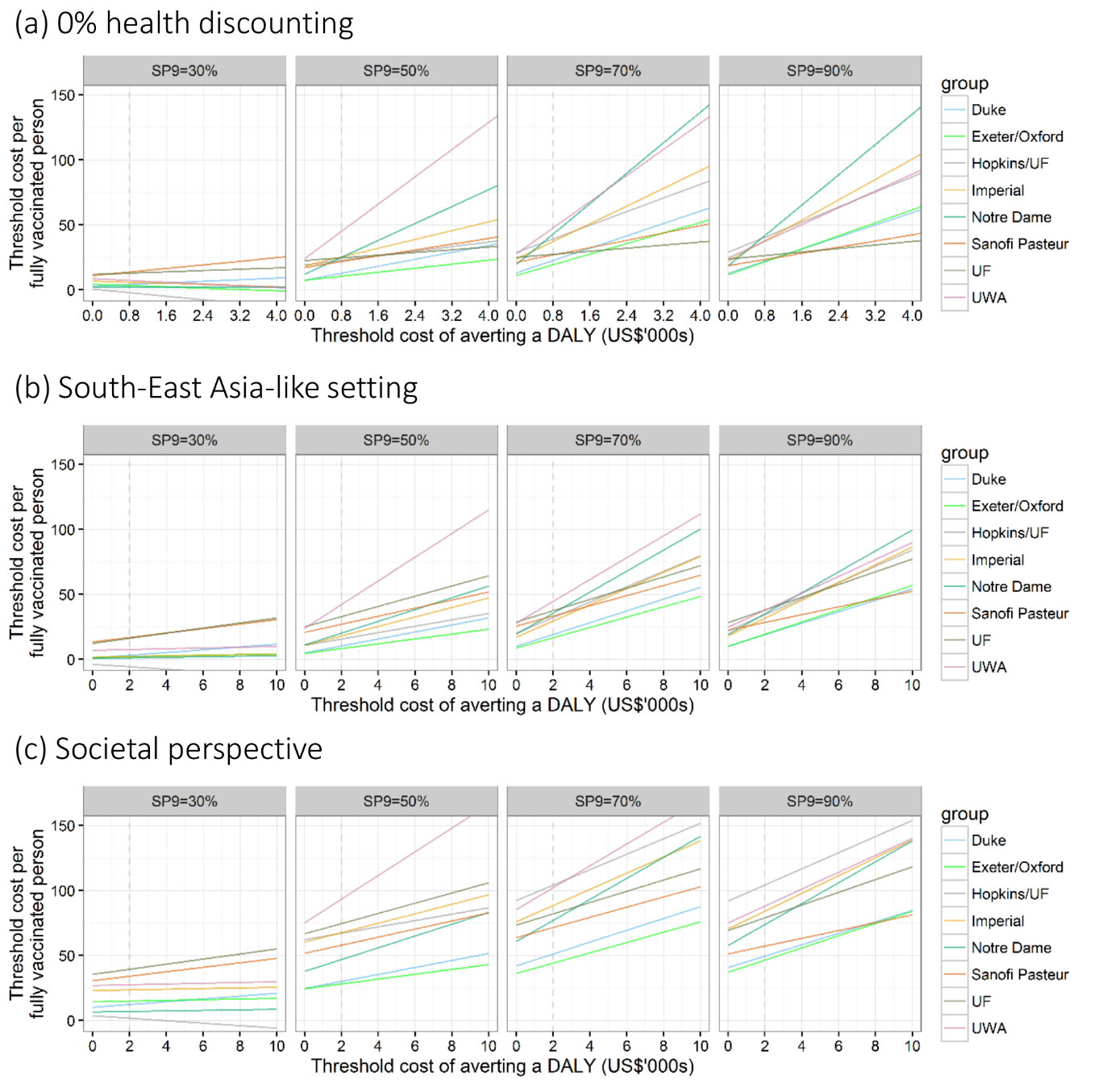


Figure K: Threshold costs per fully vaccinated person in reference to thresholds of the cost of averting a DALY for the following scenarios: (a) 0% health discounting (instead of 3%), (b) South-East Asia-like scenario (instead of Latin America-like) and (c) societal perspective (instead of health care provider). Unless otherwise stated, cost and health outcomes are calculated for 30 years after the introduction of Dengvaxia® to 9y olds with 80% coverage and without a catch-up campaign. The healthcare provider’s perspective is taken and only costs are discounted at 3%.

*Changes in economic parameters.* Figure L shows the threshold cost per vaccinated person when the costs and DALYs associated with ambulatory, hospitalised and fatal cases are varied. With a public healthcare provider perspective, there is little change to the threshold cost per vaccinated person. However, with a societal perspective the cost of dengue treatment can have a large impact on this threshold cost.


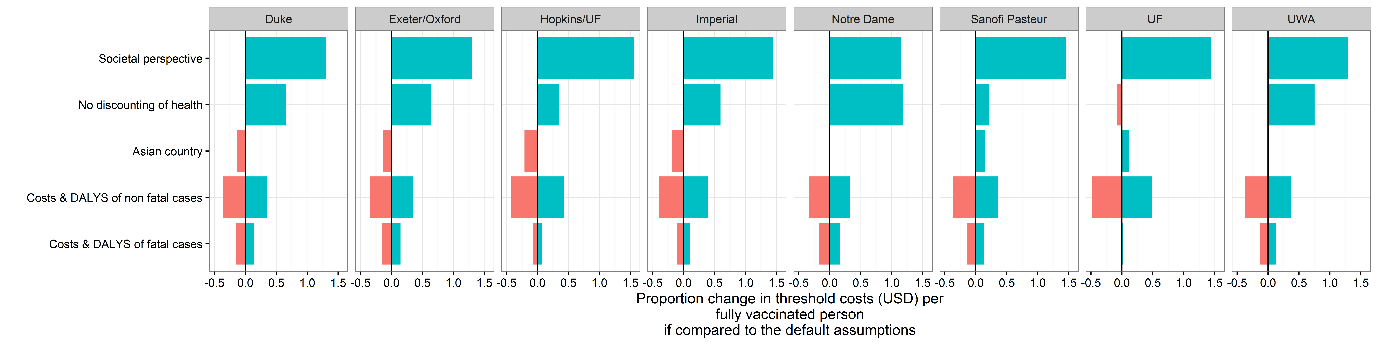

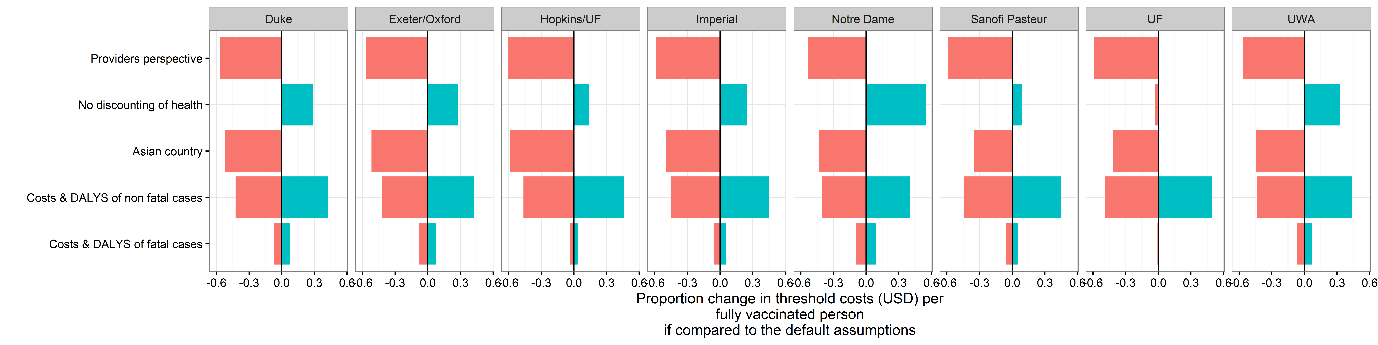


Figure L: Sensitivity analyses on the threshold costs per fully vaccinated person in a highly endemic setting (SP9=70%) and assuming the threshold costs of averting a DALY are $2000. The public sector provider perspective is taken in the upper panel and the societal perspective is taken in the lower panel.

## Tabulation of results

Table D: The proportion of hospitalised DENV cases averted within 30 years after vaccine introduction in the reference scenario. Mean and 95% range over multiple simulations are presented. This table corresponds to the data illustrated in Figure 3.

|  | SP9=10% | SP9=30% | SP9=50% | SP9=70% | SP9=90% |
| --- | --- | --- | --- | --- | --- |
| Duke | -0.04 (-0.3;0.19) | -0.03 (-0.13;0.1) | 0.07 (-0.03;0.12) | 0.17 (0.1;0.2) | 0.18 (0.08;0.24) |
| Exeter/Oxford | -0.15 (-0.28;-0.03) | -0.04 (-0.09;0.01) | 0.06 (0.04;0.08) | 0.13 (0.12;0.14) | 0.13 (0.12;0.14) |
| Hopkins/UF | -2.4 (-2.47;-2.35) | -0.16 (-0.23;-0.11) | 0.1 (0.04;0.16) | 0.2 (0.16;0.23) | 0.22 (0.2;0.24) |
| Imperial | -1.33 (-1.9;-0.63) | -0.03 (-0.1;0.02) | 0.12 (0.08;0.17) | 0.23 (0.2;0.26) | 0.25 (0.21;0.31) |
| Notre Dame | -0.23 (-0.37;-0.1) | -0.01 (-0.06;0.07) | 0.14 (0.1;0.18) | 0.22 (0.2;0.24) | 0.2 (0.15;0.23) |
| Sanofi Pasteur | -0.41 (-1.07;-0.03) | 0.15 (0.05;0.27) | 0.21 (0.12;0.31) | 0.25 (0.2;0.34) | 0.22 (0.17;0.27) |
| UF | -0.14 (-0.2;-0.08) | 0.08 (0.05;0.12) | 0.17 (0.15;0.19) | 0.17 (0.15;0.19) | 0.14 (0.12;0.16) |
| UWA | -0.18 (-0.36;0.01) | 0.08 (0.01;0.14) | 0.21 (0.19;0.23) | 0.23 (0.22;0.24) | 0.2 (0.18;0.21) |
|  |  |  |  |  |  |

Table E a-c: The number of symptomatic and hospitalised DENV cases averted per 100,000 population in the first vaccinated cohort within 30 years after vaccination. The effects of vaccination are shown for three groups: (a) the complete first vaccinated cohort, (b) those individuals who were seronegative at time of vaccination and (c) those who were seropositive at time of vaccination. The Hopkins model could not reliably follow cohorts as the age bands considered in the model were too wide.

| **(a)** | SP9=10% | SP9=30% | SP9=50% | SP9=70% | SP9=90% |
| --- | --- | --- | --- | --- | --- |
| Duke | -551 (-1180;198) | -245 (-1130;1000) | 1320 (-161;2430) | 2380 (1480;3250) | 1810 (978;2380) |
| Exeter/Oxford | -517 (-574;-461) | 86 (76;96) | 1822 (1647;1998) | 3278 (2972;3583) | 3460 (3160;3760) |
| Hopkins/UF |  |  |  |  |  |
| Imperial | -1618 (-2230;-919) | -737 (-1110;-365) | 920 (563;1262) | 2495 (2181;2808) | 2906 (2395;3420) |
| Notre Dame | -1141 (-1924;-528) | 396 (-805;1792) | 3341 (1830;4434) | 4499 (3221;5750) | 3687 (1868;5076) |
| Sanofi Pasteur | -1332 (-2065;-420) | 1232 (857;1708) | 2656 (2207;3149) | 3338 (2620;4229) | 2078 (1531;2617) |
| UF | -72 (-247;103) | 1930 (1490;2370) | 4531 (4081;4980) | 5515 (5037;5993) | 4355 (3740;4971) |
| UWA | -1737 (-2351;-1123) | 168 (-552;887) | 3833 (3037;4628) | 4023 (3589;4457) | 3120 (2697;3543) |
|  |  |  |  |  |  |

| **(b)** | SP9=10% | SP9=30% | SP9=50% | SP9=70% | SP9=90% |
| --- | --- | --- | --- | --- | --- |
| Duke | -1020 (-1940;68) | -2010 (-4180;690) | -1150 (-3120;1830) | 103 (-1750;2760) | 741 (-1930;3630) |
| Exeter/Oxford | -940 (-1041;-839) | -2048 (-2263;-1834) | -2133 (-2372;-1893) | -1556 (-1755;-1358) | -803 (-984;-622) |
| Hopkins/UF |  |  |  |  |  |
| Imperial | -3497 (-4316;-2304) | -4135 (-4505;-3776) | -3116 (-3509;-2691) | -968 (-1436;-472) | 1353 (746;1837) |
| Notre Dame | -2378 (-3687;-1331) | -2881 (-4387;-1455) | -612 (-2809;1641) | 2122 (-647;5292) | 9646 (4005;16714) |
| Sanofi Pasteur | -1626 (-2346;-608) | 138 (-476;676) | 1601 (958;2469) | 3276 (2447;5624) | 3680 (2364;4916) |
| UF | -695 (-853;-537) | 159 (-154;471) | 2237 (1844;2630) | 4329 (4004;4654) | 5788 (5345;6230) |
| UWA | -2930 (-3684;-2176) | -3325 (-4517;-2133) | -506 (-1704;692) | 698 (-600;1997) | 1634 (-190;3457) |
|  |  |  |  |  |  |

| **(c)** | SP9=10% | SP9=30% | SP9=50% | SP9=70% | SP9=90% |
| --- | --- | --- | --- | --- | --- |
| Duke | 1670 (1030;2210) | 3730 (2100;5390) | 4480 (2180;6050) | 4240 (2140;5880) | 2440 (1140;3170) |
| Exeter/Oxford | 1196 (1077;1316) | 2862 (2590;3135) | 3568 (3240;3896) | 3387 (3103;3672) | 2349 (2179;2520) |
| Hopkins/UF |  |  |  |  |  |
| Imperial | 1841 (1495;2229) | 5574 (5170;6084) | 7389 (6869;7993) | 7259 (6432;7897) | 4858 (3843;5872) |
| Notre Dame | 3354 (576;5965) | 7714 (5987;9896) | 9289 (7627;10618) | 8158 (6962;9875) | 5153 (3325;6617) |
| Sanofi Pasteur | 1126 (500;1754) | 3231 (2850;3678) | 3566 (3054;3946) | 3370 (2638;3765) | 1905 (1415;2403) |
| UF | 2691 (2176;3207) | 6641 (6141;7142) | 8435 (8201;8669) | 8244 (7943;8545) | 5522 (5025;6020) |
| UWA | 5661 (4372;6951) | 9643 (8233;11054) | 10237 (9020;11455) | 8253 (7664;8843) | 4286 (3994;4577) |
|  |  |  |  |  |  |

Table F a-d: The proportion of symptomatic and hospitalised DENV cases averted in the 30 years after vaccine introduction for low (a) to very high (d) transmission intensity. Each point represents a model realisation at a given age of vaccine introduction (columns).

| **(a)** | 9 | 10 | 11 | 12 | 13 | 14 | 15 | 16 | 17 | 18 |
| --- | --- | --- | --- | --- | --- | --- | --- | --- | --- | --- |
| Duke | -0.03 | -0.02 | -0.02 | 0.01 | 0.01 | 0.02 | 0.03 | 0.04 | 0.05 | 0.05 |
| Exeter/Oxford | -0.04 |  |  | 0.02 |  |  |  | 0.06 |  |  |
| Hopkins/UF | -0.16 | -0.13 | -0.09 | -0.07 | -0.04 | 0 |  | 0.06 |  |  |
| Imperial | -0.03 | -0.01 | 0.02 | 0.04 | 0.06 | 0.08 | 0.09 | 0.11 | 0.12 | 0.14 |
| Notre Dame | -0.01 |  |  |  |  |  |  | 0.08 |  |  |
| Sanofi Pasteur | 0.15 |  | 0.18 |  | 0.21 |  | 0.23 |  | 0.25 |  |
| UF | 0.08 | 0.1 | 0.09 | 0.12 | 0.13 | 0.12 | 0.16 | 0.17 | 0.19 | 0.18 |
| UWA | 0.08 |  |  |  |  |  |  | 0.16 |  |  |

| **(b)** | 9 | 10 | 11 | 12 | 13 | 14 | 15 | 16 | 17 | 18 |
| --- | --- | --- | --- | --- | --- | --- | --- | --- | --- | --- |
| Duke | 0.07 | 0.08 | 0.09 | 0.1 | 0.1 | 0.11 | 0.11 | 0.12 | 0.12 | 0.13 |
| Exeter/Oxford | 0.06 |  |  | 0.1 |  |  |  | 0.1 |  |  |
| Hopkins/UF | 0.1 | 0.12 | 0.14 | 0.16 | 0.18 | 0.21 |  | 0.22 |  |  |
| Imperial | 0.12 | 0.14 | 0.16 | 0.17 | 0.18 | 0.19 | 0.2 | 0.21 | 0.21 | 0.22 |
| Notre Dame | 0.14 |  |  |  |  |  |  | 0.18 |  |  |
| Sanofi Pasteur | 0.21 |  | 0.23 |  | 0.25 |  | 0.26 | 0.26 | 0.26 |  |
| UF | 0.17 | 0.16 | 0.17 | 0.17 | 0.18 | 0.18 | 0.17 | 0.18 | 0.19 | 0.2 |
| UWA | 0.21 |  |  |  |  |  |  | 0.25 |  |  |

| **(c)** | 9 | 10 | 11 | 12 | 13 | 14 | 15 | 16 | 17 | 18 |
| --- | --- | --- | --- | --- | --- | --- | --- | --- | --- | --- |
| Duke | 0.17 | 0.16 | 0.17 | 0.16 | 0.16 | 0.16 | 0.16 | 0.15 | 0.14 | 0.14 |
| Exeter/Oxford | 0.13 |  |  | 0.13 |  |  |  | 0.1 |  |  |
| Hopkins/UF | 0.2 | 0.22 | 0.23 | 0.23 | 0.23 | 0.24 |  | 0.21 |  |  |
| Imperial | 0.23 | 0.23 | 0.23 | 0.23 | 0.23 | 0.23 | 0.22 | 0.22 | 0.21 | 0.2 |
| Notre Dame | 0.22 |  |  |  |  |  |  | 0.15 |  |  |
| Sanofi Pasteur | 0.25 |  | 0.25 |  | 0.25 |  | 0.24 | 0.23 | 0.22 |  |
| UF | 0.17 | 0.18 | 0.17 | 0.17 | 0.16 | 0.15 | 0.16 | 0.16 | 0.15 | 0.14 |
| UWA | 0.23 |  |  |  |  |  |  | 0.24 |  |  |

| **(d)** | 9 | 10 | 11 | 12 | 13 | 14 | 15 | 16 | 17 | 18 |
| --- | --- | --- | --- | --- | --- | --- | --- | --- | --- | --- |
| Duke | 0.18 | 0.16 | 0.15 | 0.13 | 0.11 | 0.1 | 0.09 | 0.07 | 0.06 | 0.06 |
| Exeter/Oxford | 0.13 |  |  | 0.09 |  |  |  | 0.05 |  |  |
| Hopkins/UF | 0.22 | 0.2 | 0.18 | 0.16 | 0.14 | 0.12 | NA | 0.08 |  |  |
| Imperial | 0.25 | 0.23 | 0.21 | 0.19 | 0.17 | 0.15 | 0.13 | 0.11 | 0.09 | 0.08 |
| Notre Dame | 0.2 |  |  |  |  |  |  | 0.06 |  |  |
| Sanofi Pasteur | 0.22 |  | 0.19 |  | 0.16 |  | 0.13 | 0.12 | 0.1 |  |
| UF | 0.14 | 0.13 | 0.11 | 0.09 | 0.08 | 0.08 | 0.07 | 0.06 | 0.06 | 0.06 |
| UWA | 0.2 |  |  |  |  |  |  | 0.1 |  |  |

Table G: Threshold costs (in US$) per fully vaccinated person in reference to thresholds of the cost of averting a DALY (assumed to be 2000US$ here). Cost and health outcomes are calculated for 30 years after the introduction of Dengvaxia® to 9y olds with 80% coverage and without a catch-up campaign. The healthcare provider’s perspective is taken and both health and costs are discounted at 3%.

|  | SP9=30% | SP9=50% | SP9=70% | SP9=90% |
| --- | --- | --- | --- | --- |
| Duke | 5 (1;9) | 13 (9;16) | 22 (20;25) | 22 (18;24) |
| Exeter/Oxford | 5 (-2;12) | 11 (8;15) | 19 (16;21) | 21 (19;23) |
| Hopkins/UF | -1 (-5;3) | 24 (16;31) | 41 (33;46) | 41 (38;46) |
| Imperial | 7 (18;-2) | 26 (37;16) | 36 (46;27) | 36 (45;26) |
| Notre Dame | 2 (-2;10) | 21 (16;27) | 36 (32;39) | 35 (28;39) |
| Sanofi Pasteur | 14 (6;24) | 24 (15;34) | 29 (23;40) | 23 (18;28) |
| UF | 16 (13;19) | 30 (27;34) | 34 (29;38) | 33 (29;38) |
| UWA | 9 (-1;19) | 42 (34;50) | 44 (37;52) | 38 (30;45) |

## Including Y2 LTFU data

### Inclusion without additional fit

Data from the second year of passive follow up (LTFUY2 – the 4^th^ year after the start of the trial) have just become publically available. Using the models parameterised to data from the first three years of the trial we predicted a fourth year in the trial setting and compared our results to the trial observations (Figure M). Given overall dengue attack rates in the trial varied substantially between Y3 and Y4, we present the comparison with model output in terms of the relative risk of hospitalised dengue by age group, comparing the vaccine and control arms. Most models predict rather subtle change in the relative risks for hospitalisation due to dengue from LTFUY1 to LTFUY2. While this led to an improved fit of the models for LTFUY2 data in 2-5y olds and 12-14 year olds if compared with LTFUY1 data, no model reproduced the increased risk of hospitalisation in 6-11 years old vaccinees in LTFUY2. Although UF reports relatively high mean attack rates, these are strongly affected by a small number of outliers: median attack rates are closer to the empirical data (from left to right as in Figure L: 2-5y [0.008, 0.010], 6-11y [0.008, 0.006], 12-14y [0.007, 0.004]).


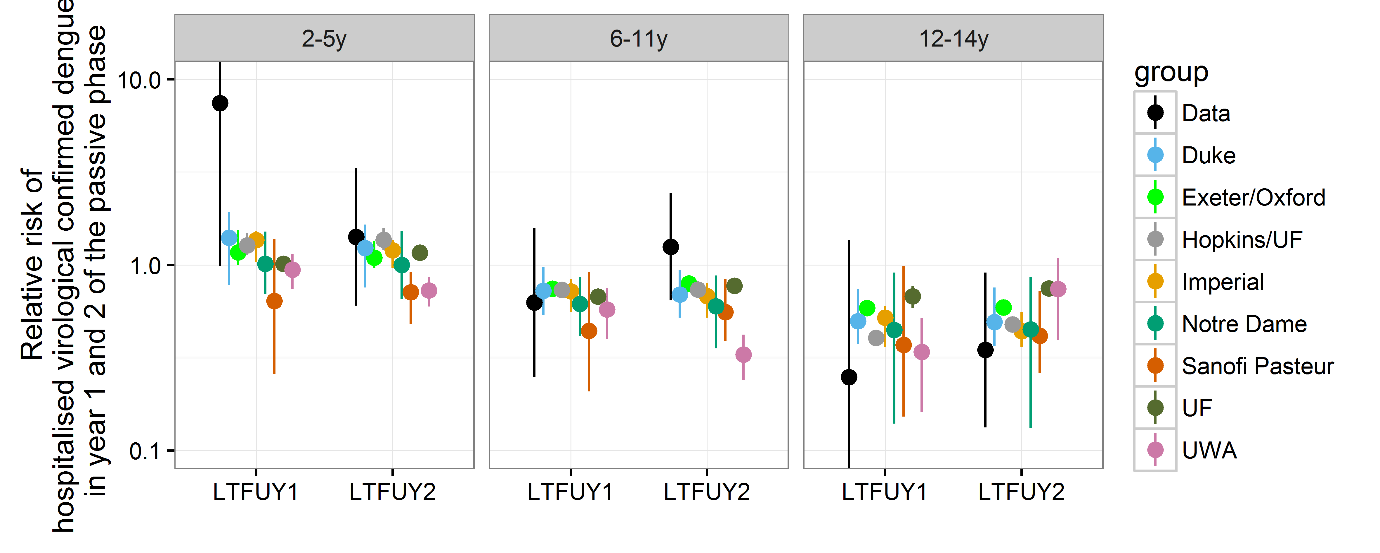


Figure M: predictions for year 2 of the long term follow up (LTFUY2). The relative risks for dengue hospitalisation in LTFUY1 and LTFUY2 are shown. Note that while the mean attack rate among vaccinees 2-5 years old in the UF model is lower than that among controls the paired posterior samples show an increased relative risk among vaccinees. For the data (black) dots report mean estimates and error bars 95% binomial confidence intervals. For model predictions dots report mean estimates and error bars the 95% range of simulations.

### Inclusion with fitting

Four groups also included the new LTFUY2 data into their model fits. For the Sanofi Pasteur model this meant including disaggregated data of years 1 to 3 and aggregated data from Y4. Furthermore, the Sanofi Pasteur model was adapted to include increased efficacy with severity and differential efficacy with age.

Including the additional trial data led to very minor changes in parameter estimates for all models compared with estimates obtained from fitting to years 1 to 3 alone. The increase in relative risk for hospitalisation in the 6-11 year age group in LTFUY2 could not be reproduced by any of the models (Figure N). Potential ways to enable enough flexibility of the vaccine model to reproduce this increased risk could include waning immunity of seropositive individuals. This in turn would lead to more pessimistic long-term predictions of the impact of Dengvaxia®. Data from another year of follow-up is needed to establish if these trends continue, thus potentially requiring revision of the model of vaccine action used here.


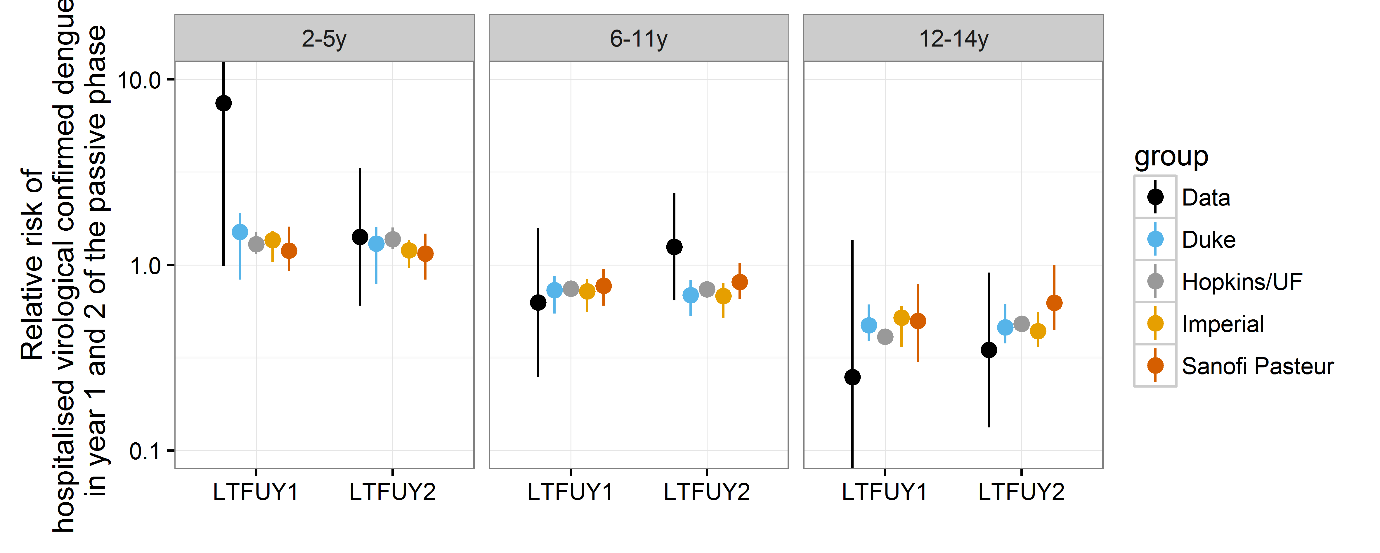
Figure N: predictions resulting from model fits including year 2 of the long term follow up (LTFUY2). The relative risks for dengue hospitalisation in LTFUY1 and LTFUY2 are shown. For the data (black) dots report mean estimates and error bars 95% binomial confidence intervals. For model predictions dots report mean estimates and error bars the 95% range of simulations.

## References

1. Nagao Y, Koelle K. Decreases in dengue transmission may act to increase the incidence of dengue hemorrhagic fever. Proc Natl Acad Sci. 2008;105: 2238–2243. doi:10.1073/pnas.0709029105

2. Lourenco J, Recker M. Natural, Persistent Oscillations in a Spatial Multi-Strain Disease System with Application to Dengue. Pascual M, editor. PLoS Comput Biol. Public Library of Science; 2013;9: e1003308. doi:10.1371/journal.pcbi.1003308

3. Hladish TJ, Pearson CAB, Chao DL, Rojas DP, Recchia GL, Gómez-Dantés H, et al. Projected Impact of Dengue Vaccination in Yucatán, Mexico. Carvalho MS, editor. PLoS Negl Trop Dis. 2016;10: e0004661. doi:10.1371/journal.pntd.0004661

4. Ferguson NM, Rodriguez-Barraquer I, Dorigatti I, Mier-y-Teran-Romero L, Laydon DJ, Cummings DAT. Benefits and risks of the Sanofi-Pasteur dengue vaccine: Modeling optimal deployment. Science (80- ). 2016;353: 1033–1036. doi:10.1126/science.aaf9590

5. Rodriguez-Barraquer I, Mier-y-Teran-Romero L, Schwartz IB, Burke DS, Cummings DAT. Potential opportunities and perils of imperfect dengue vaccines. Vaccine. 2014;32: 514–20. doi:10.1016/j.vaccine.2013.11.020

6. Rodríguez-Barraquer I, Buathong R, Iamsirithaworn S, Nisalak A, Lessler J, Jarman RG, et al. Revisiting Rayong: Shifting Seroprofiles of Dengue in Thailand and Their Implications for Transmission and Control. Am J Epidemiol. 2014;179: 353–360. doi:10.1093/aje/kwt256

7. Perkins TA, Reiner RC, ten Bosch QA, Espana G, Verma A, Liebman KA, et al. Statistical and biological uncertainties associated with vaccine efficacy estimates and their implications for dengue vaccine impact projections. bioRxiv. 2016; doi: http://dx.doi.org/10.1101/082396.

8. Coudeville L, Baurin N, Vergu E. Estimation of parameters related to vaccine efficacy and dengue transmission from two large phase III studies. Vaccine. 2015; 1–9. doi:10.1016/j.vaccine.2015.11.023

9. Karl S, Halder N, Kelso JK, Ritchie S a., Milne GJ. A spatial simulation model for dengue virus infection in urban areas. BMC Infect Dis. 2014;14: 447. doi:10.1186/1471-2334-14-447

10. Hadinegoro SR, Arredondo-García JL, Capeding MR, Deseda C, Chotpitayasunondh T, Dietze R, et al. Efficacy and Long-Term Safety of a Dengue Vaccine in Regions of Endemic Disease. N Engl J Med. 2015; 150727090428004. doi:10.1056/NEJMoa1506223

11. Martelli CMT, Siqueira JB, Parente MPPD, Zara AL de SA, Oliveira CS, Braga C, et al. Economic Impact of Dengue: Multicenter Study across Four Brazilian Regions. PLoS Negl Trop Dis. 2015;9: e0004042. doi:10.1371/journal.pntd.0004042

12. Edillo FE, Halasa YA, Largo FM, Erasmo JN V, Amoin NB, Alera MTP, et al. Economic cost and burden of dengue in the Philippines. Am J Trop Med Hyg. 2015;92: 360–366. doi:10.4269/ajtmh.14-0139

13. Villar L, Dayan GH, Arredondo-García JL, Rivera DM, Cunha R, Deseda C, et al. Efficacy of a Tetravalent Dengue Vaccine in Children in Latin America. N Engl J Med. 2015;372: 113–123. doi:10.1056/NEJMoa1411037

14. Capeding MR, Tran NH, Hadinegoro SRS, Ismail HIHM, Chotpitayasunondh T, Chua MN, et al. Clinical efficacy and safety of a novel tetravalent dengue vaccine in healthy children in Asia: A phase 3, randomised, observer-masked, placebo-controlled trial. Lancet. 2014;384: 1358–1365. doi:10.1016/S0140-6736(14)61060-6

15. World Health Organization. The Global Burden of Disease: 2004 Update. 2008. doi:10.1038/npp.2011.85

16. Newall AT, Jit M, Hutubessy R. Are current cost-effectiveness thresholds for low- and middle-income countries useful? Examples from the world of vaccines. PharmacoEconomics. 2014. pp. 525–531. doi:10.1007/s40273-014-0162-x

17. Marseille E, Larson B, Kazi DS, Kahn JG, Rosen S. Thresholds for the cost-effectiveness of interventions: alternative approaches. Bull World Health Organ. 2015;93: 118–24. doi:10.2471/BLT.14.138206

18. Jit M, Brisson M, Portnoy A, Hutubessy R. Cost-effectiveness of female human papillomavirus vaccination in 179 countries: a PRIME modelling study. Lancet Glob Heal. World Health Organization; 2014;2: e406–e414. doi:10.1016/S2214-109X(14)70237-2

19. Sinha A, Levine O, Knoll MD, Muhib F, Lieu TA. Cost-effectiveness of pneumococcal conjugate vaccination in the prevention of child mortality: an international economic analysis. Lancet. 2007;369: 389–396. doi:10.1016/S0140-6736(07)60195-0

20. Rheingans RD, Antil L, Dreibelbis R, Podewils LJ, Bresee JS, Parashar UD. Economic costs of rotavirus gastroenteritis and cost-effectiveness of vaccination in developing countries. J Infect Dis. 2009;200 Suppl: S16–S27. doi:10.1086/605026

21. Shepard DS, Halstead SB. Dengue (with notes on yellow fever and Japanese encephalitis). Disease Control Priorities for Developing Countries. 1993. pp. 303–320.

22. Kim S-Y, Goldie SJ, Salomon JA. Cost-effectiveness of Rotavirus vaccination in Vietnam. BMC Public Health. 2009;9: 29. doi:10.1186/1471-2458-9-29

23. Ayieko P, Griffiths UK, Ndiritu M, Moisi J, Mugoya IK, Kamau T, et al. Assessment of Health Benefits and Cost-Effectiveness of 10-Valent and 13-Valent Pneumococcal Conjugate Vaccination in Kenyan Children. PLoS One. 2013;8: e67324. doi:10.1371/journal.pone.0067324
